# Synthetic lethality of drug-induced polyploidy and BCL-2 inhibition in lymphoma

**DOI:** 10.1038/s41467-023-37216-2

**Published:** 2023-03-18

**Authors:** Ana Portelinha, Mariana da Silva Ferreira, Tatiana Erazo, Man Jiang, Zahra Asgari, Elisa de Stanchina, Anas Younes, Hans-Guido Wendel

**Affiliations:** 1 Cancer Biology & Genetics Program, Memorial Sloan-Kettering Cancer Center, New York, NY 10065 USA; 2 Department of Medicine Lymphoma Service Memorial Sloan-Kettering Cancer Center, New York, NY 10065 USA; 3 Antitumor Assessment Core, Memorial Sloan Kettering Cancer Center, New York, NY USA; 4 Present Address: AstraZeneca, Medimmune Way, Gaithersburg, MD USA

**Keywords:** Targeted therapies, B-cell lymphoma, B-cell lymphoma

## Abstract

Spontaneous whole genome duplication and the adaptive mutations that disrupt genome integrity checkpoints are infrequent events in B cell lymphomas. This suggests that lymphomas might be vulnerable to therapeutics that acutely trigger genomic instability and polyploidy. Here, we report a therapeutic combination of inhibitors of the Polo-like kinase 4 and BCL-2 that trigger genomic instability and cell death in aggressive lymphomas. The synthetic lethality is selective for tumor cells and spares vital organs. Mechanistically, inhibitors of Polo-like kinase 4 impair centrosome duplication and cause genomic instability. The elimination of polyploid cells largely depends on the pro-apoptotic BAX protein. Consequently, the combination of drugs that induce polyploidy with the BCL-2 inhibitor Venetoclax is highly synergistic and safe against xenograft and PDX models. We show that B cell lymphomas are ill-equipped for acute, therapy-induced polyploidy and that BCL-2 inhibition further enhances the removal of polyploid lymphoma cells.

## Abstract (web summary)

Genomic instability occurs infrequently in in diffuse large B cell lymphoma (DLBCL), suggesting a therapeutic vulnerability. Here, the authors identify a synergistic combination between the induction of polyploidy by a PLK4 inhibitor and a BCL-2 inhibitor in DLBCL.

## Introduction

Whole genome duplication (WGD) is a rare event in indolent and even in aggressive types of B cell lymphomas. For example, diffuse large B cell lymphoma (DLBCL) or transformed follicular lymphomas (tFLs) show frequencies of polyploidy of less than 3% with a modest impact on prognosis^1–4^. Consistently, a new genomics-centered DLBCL classification identifies a small (8.7%) group of Aneuploid/*TP53* mutant (“AP”) tumors suggesting that genomic instability and the mutations that enable the survival of such unstable cells are infrequent drivers of DLBCL^5^. In contrast, WGD is a frequent event in solid tumors that drives tumor genome evolution and contributes to disease progression, resistance, metastasis, and poor clinical outcomes^6–8^. WGD represents a major cell stress that tumor cells must adapt to by disabling critical proliferation and genome checkpoints like TP53 and RB1^9–12^. These adaptations are hallmarks of most solid cancers, by contrast, they are rare events in lymphomas suggesting they are unprepared and vulnerable to therapeutics that can cause genomic instability^13–20^.

Polo-like kinase 4 (PLK4) is a self-regulatory serine/threonine kinase that positively regulates centriole duplication, and its loss leads to deficient centriole numbers^21–26^. Inactivation of PLK4 causes genomic instability, aneuploidy, and polyploidy in proliferating cancer cells^27–31^. In solid tumors such as breast and lung cancer, PLK4 inhibition leads to ongoing endoreduplication with large and polyploid (up to 32n DNA content) cells that eventually undergo cell death or senesce^28,32,33^. PLK4 is highly expressed in many cancers, including B cell lymphomas, but also expressed in normal cells, and its expression alone does not appear to indicate a functional requirement^28,31,34^. CFI-400945 is a potent PLK4 inhibitor that has entered clinical testing in solid tumors and myeloid leukemia (NCT03624543; NCT03385655; and NCT01954316)^28,35^. Despite extensive study, the PLK4 selectivity of CFI-400945 remains a topic of debate^4,28,36–38^ by the fact that may also inhibit Aurora B kinase at ~40-fold higher concentrations than PLK4 and the resultant cytokinesis defects may further contribute to CFI-induced polyploidy^28,39^. Given the infrequent occurrence of spontaneous polyploidy in lymphomas, we wondered if they would be especially sensitive to this therapeutic mechanism.

Apoptosis is a key mechanism to eliminate genomically unstable and polyploid tumor cells. The anti-apoptotic BCL-2 protein is frequently overexpressed, amplified, and translocated in several forms of B cell lymphoma^40–43^. BCL-2 is highly expressed in nearly all follicular lymphomas and in 30% of aggressive, diffuse large B cell and mantle cell lymphomas and can potentially protect polyploid lymphoma cells from cell death^41,42,44^. Venetoclax is a highly selective, platelet-sparing, and clinically approved BCL-2 inhibitor^45,46^. Mechanistically, venetoclax acts as a BH3-mimetic and impairs BCL-2 binding to pro-apoptotic proteins, including BAX, BAK, and other BH3-only proteins. Venetoclax effects may cascade beyond BCL-2 as BH3 proteins are freed to bind and block other anti-apoptotic proteins like MCL-1 and BCL-xL^47^. Moreover, recent work has revealed additional BCL-2 independent venetoclax effects on tumor cell metabolism that trigger broader cell stress responses^48^.

Here we show, the potential for venetoclax to specifically augment sensitivity to drug-induced genomic instability and polyploidy in B cell lymphomas without increased toxicity in healthy subjects.

## Results

### Defining the activity of CFI-400945 against lymphoma cell lines

**Fig. 1:**
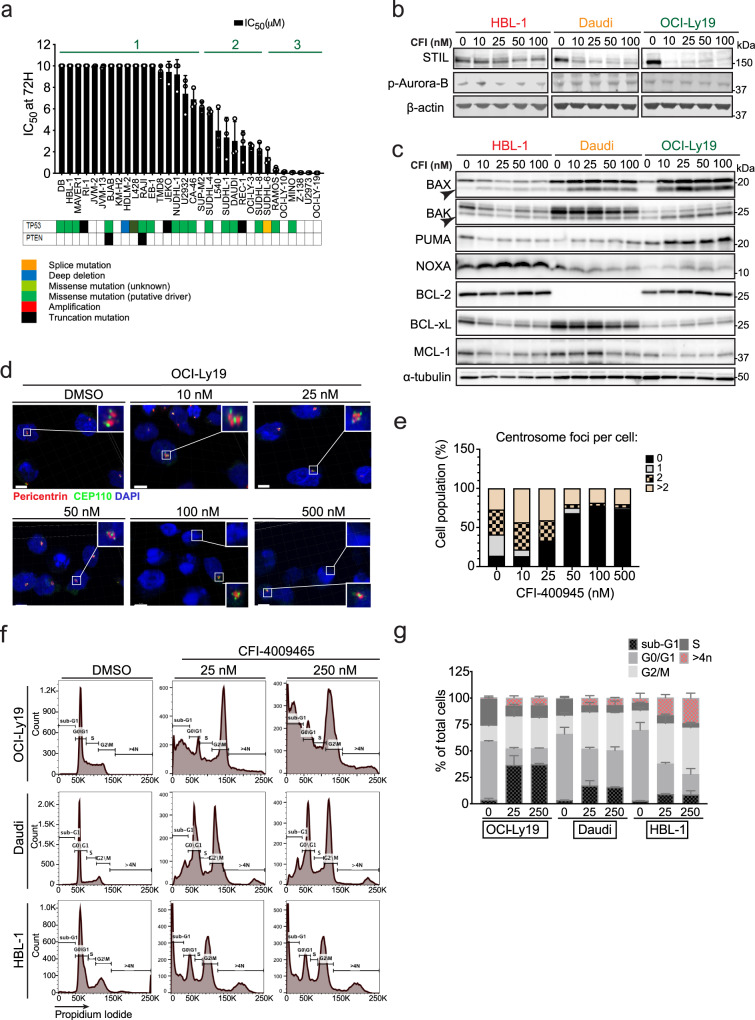
The PLK4 inhibitor CFI-400945 causes polyploidy and cell death in lymphoma cells. **a** CFI half maximal inhibitory concentration (IC_50_) values in a panel of cell lines representing different subtypes of lymphoma treated with CFI for 72 h. Cell viability was measured by CellTiter-Glow assay. Each bar represents the mean ± SD of 3 independent experiments, each time in triplicate. Responses to CFI treatment is categorized as resistant (1), intermediate (2), and Sensitive (3) cell lines. **b** Western blot analysis of total STIL and Aurora B phosphorylation levels in CFI-resistant and sensitive cell lines treated with increasing concentration of CFI for 72 h. β-actin was used as a loading control. **c** Western blot analysis of anti- and pro-apoptotic Bcl-2 family proteins. Sensitive cell lines exhibit overexpression of pro-apoptotic proteins after treatment with CFI at the indicated doses. α-tubulin was used as a loading control. **d** Representative confocal images of OCI-Ly19 cells treated with dimethyl sulfoxide (DMSO) (0.1%) or with the indicated doses of CFI for 72 h. Treatment with 10–50 nM of CFI causes centrosome amplification, while 500 nM leads to complete failure in duplication. Cells were fixed and stained for pericentrin and CEP110. Insets are 4X magnified. Scale bar, 5 μm. Images are representative of two independent experiments. A minimum of three z-stack images were captured under the same condition. **e** Stacked bar graph of the percentage of cells with 0, 1, 2, or over 2 centrosomes per cell in OCI-Ly19 cells treated with CFI. Values represent the experiment shown in panel **c**. **f** Representative flow cytometric analyses of cell cycle distribution by propidium iodide (PI) staining in CFI sensitive (OCI-Ly19), intermediate (Daudi), and resistant (HBL-1) cell lines after 72 h of CFI treatment (25 or 250 nM) or control (DMSO 0.1%). Data represent two independent experiments (*n* = 2). **g** Stacked bar graph representing the cell-cycle distribution of PI staining in three lymphoma cell lines treated with either control (DMSO, 0.1%) or CFI (25 or 250 nM) for 72 h. CFI induced a specific G2/M arrest and polyploidy in the resistant cell lines and an increase in the sub-G1 population on the CFI-sensitive cell lines. Data were represented as mean percentage ± SD (*n* = 2 independent experiments). Each immunoblot results in the representative of at least three repeats. Source data for panels **a**–**e**, **g** are provided as a Source Data file.

We screened a panel of 32 B cell lymphoma cell lines for sensitivity to the PLK4 inhibitor CFI-400945. Cell lines were chosen to represent a broad spectrum of B cell lymphoma subtypes, including ABC or GCB-DLBCL, transformed FL, mantle cell, Burkitt’s, Hodgkin’s, anaplastic B cell lymphoma, and to reflect a spectrum of the most commonly occurring mutations (Suppl. Figure 1a). We determined concentrations of CFI-400945 (form here on “CFI”) that induced 50% cell death (IC_50_) in 72 h drug exposures. We identified three broad groups of highly sensitive (IC_50_ < 0.05 µM), intermediate (IC_50_ 1–6 μM), and CFI-resistant lymphoma cell lines (IC_50_ > 10 μM) (Fig. 1a). The groups did not reflect specific subtypes, PLK4 expression levels (Suppl. Fig. 1b) or mutation patterns although we noticed that four of the five most sensitive lines were wildtype for TP53 and this is consistent with evidence that PLK4 inhibition can activate a P53-dependent response^30^ (Fig. 1a). PTEN mutations had been implicated in CFI sensitivity in breast cancer^49^; however, PTEN lesions were too rare to evaluate in this lymphoma panel.

Next, we performed immunoblots on CFI-treated cells to understand the molecular changes following CFI treatment, and we focused on indicators of target engagement and cell death proteins. The selectivity of CFI for PLK4 has been a topic of much discussion, and Aurora B kinase has been implicated as an additional CFI target at higher concentrations^27,28^. We confirmed PLK4 inhibition by CFI as indicated by the loss of the STIL protein, which depends on PLK4 for stability^50–52^. Additionally, we measured auto-phosphorylation of Aurora B kinase, and we observed modest inhibition only in Daudi cells at 100 nM, while other lymphoma cell lines showed no evidence of Aurora inhibition at this high concentration (Fig. 1b). We noticed that CFI treatment induced pro-apoptotic proteins especially BAX, BAK, PUMA, and NOXA in the CFI-sensitive cells (OCI-Ly19 and Z-138) and less in intermediate (Daudi, SU-DHL-4) or the CFI-resistant cells (HBL-1, DB); we observed no change in anti-apoptotic BCL-2 family proteins BCL-2, BCL-xL, MCL-1 (Fig. 1c and Suppl. Fig. 1c)

We then used confocal microscopy on CFI-sensitive cells to evaluate the molecular effects of PLK4 inhibition. We chose OCI-Ly19 cells which are the most sensitive line in our panel (IC_50_ ~30 nM after 72 h exposure). While low doses (10 nM and up to 25 nM) cause centrosome overduplication in Ly19 cells, higher doses (100 nM and up to 500 nM) result in centrosome loss (Fig. 1d, e). This bi-modal effect has been reported in breast cancer cells where partial inhibition of PLK4 dimeric complexes was shown to impair degradation of PLK4, resulting in a paradoxical increase of overall PLK4 activity, consistent with prior reports; we observed centrosome loss indicating complete inhibition beginning at 50 nM of CFI (Fig. 1e)^25,28,53^. We also analyzed CFI effects on cell cycle distribution in pairs of sensitive (OCI-Ly19 and Z-138), intermediate sensitive (Daudi and SU-DHL-4), and CFI-resistant cells (HBL-1 and DB). The sensitive cells showed efficient cell death induction with a prominent sub-G1 population and no polyploidy; on the other hand, CFI-resistant cells showed accumulation of a polyploid (4n) population consistent with G2M arrest and without significant apoptosis, the two intermediate cell lines showed a mixed phenotype with fewer polyploid cells and limited cell death (Fig. 1f, g and Suppl. Fig. 1d–f). Further, Annexin V-FITC/PI staining readily confirmed these differences in cell death induction among the same sensitive, intermediate, and resistant pairs of cell lines (Suppl. Fig. 2a).

We further tested centrinone as an alternate and potentially more selective PLK4 inhibitor. However, centrinone is not used in vivo or clinically^54,55^. Centrinone showed the same pattern of sensitivity and resistance with lymphoma cell lines that showed nanomolar sensitivity to CFI being similarly sensitive to centrinone (e.g., Z-138 and OCI-Ly19) (Suppl. Fig. 2b). We further saw analogous effects on apoptosis induction and G2M arrest in sensitive cells and the emergence of polyploid populations in resistant lines (Suppl. Figs. 2c, 1d). Similar patterns of cell death induction by Annexin-FITC/PI staining were also observed at higher concentrations reflecting its lower molar potency (Suppl. Fig. 1e). Together, these data reveal a pattern of efficient cell death induction in CFI and centrinone-sensitive cells, while resistant lymphoma cells showed an accumulation of polyploid cells that failed to undergo apoptosis.

### BAX determines the cellular response to CFI-400945

**Fig. 2:**
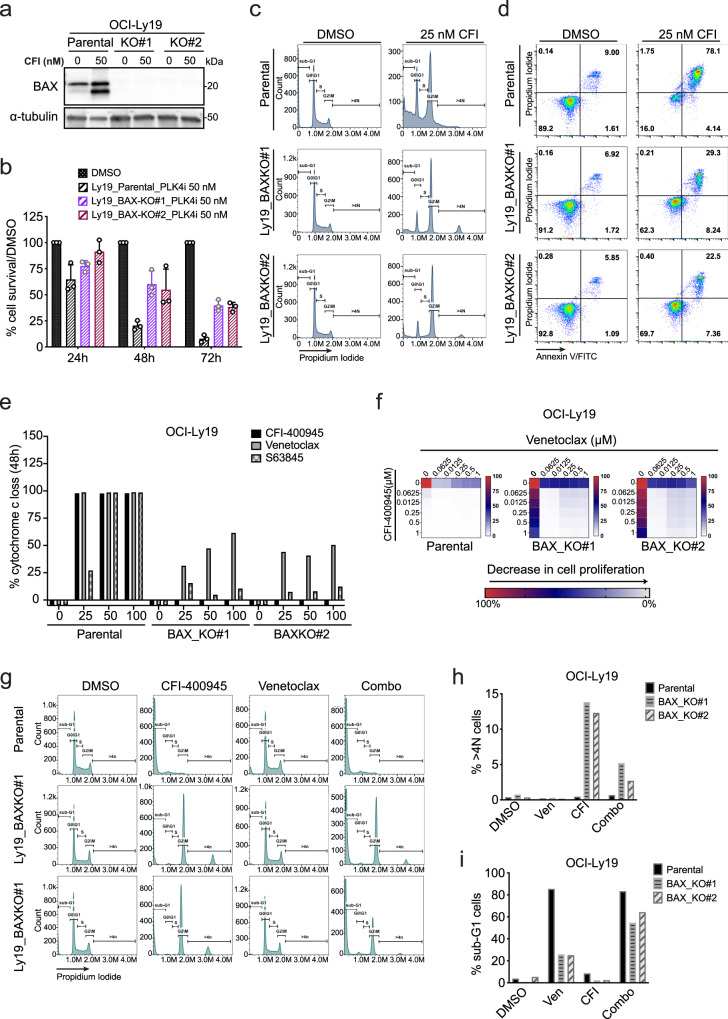
The elimination of polyploid lymphoma cells depends on BAX. **a** Western blot confirmation of BAX protein depletion in OCI-Ly19 knockout clones (BAXKO#1 and BAXKO#2) treated with CFI (25 nM) or with control (DMSO, 0.1%). Each immunoblot result is representative of at least three repeats. **b** Cell proliferation of OCI-Ly19 and isogenic BAXKO clones after 24, 48, and 72 h of treatment with 50 nM of CFI or with DMSO by CellTiter-Glo luminescent assay and normalized to DMSO-treated cells at each time point. Values represent means ± SD of three independent experiments. **c** Representative flow cytometry analysis on cell cycle progression and **d** apoptosis within OCI-Ly19 isogenic cell lines upon 72 h treatment with CFI (25 nM) or control (DMSO 0.1%). **e** Direct mitochondrial response by iBH3 assay in OCI-Ly19 parental and BAX-deficient clones pre-treated for 48 h with venetoclax (BCL-2 inhibitor), S63845 (MCL-1 inhibitor), or CFI. **f** Drug matrix heatmap shows synergistic interaction of CFI with venetoclax in OCI-Ly19 isogenic cell lines. Heatmap grids correspond to the mean of 3 independent experiments by the CellTiter-Glo assay. Cell viability values are represented in a heatmap colored from red (0% inhibition) or blue to white (50–100% inhibition). Values represent the average of three independent dose-titration curves (*n* = 3). **g** Representative flow cytometry analysis on cell cycle progression with PI staining in OCI-Ly19 isogenic cell lines treated with CFI (25 nM), Venetoclax (25 nM), or with the combination of both drugs (Combo) for 72 h treatment. Flow cytometry analysis results in the representative of at least three repeats. **h** Graphical representation of the percentage of >4 N cells or **i** sub-G1 cells for OCI-Ly19 cell lines shown in panel **g**. Ven Venetoclax, Combo combination of CFI and Venetoclax. Source data for panels **a**, **b**, **e**, **f**, **h**, **i** are prov**i**ded as a Source Data file.

It is known that CFI treatment activates the P53 checkpoint^30^ and our data indicate induction of the P53 target, BAX, is a feature of CFI-sensitive cell lines (Fig. 1c and Suppl. Fig. 1b). To better understand the role of BAX in CFI sensitivity we generated isogenic BAX wild-type and BAX-deficient clones from two PLK4 sensitive lymphoma lines (OCI-LY19 and Z-138). We confirmed complete loss of BAX by immunoblot and survival assays (Fig. 2a and Suppl. Fig. 3a). BAX loss was sufficient to confer CFI resistance in both OCI-Ly19 and Z-138 lines (Fig. 2b and Suppl. Fig. 3b), and while the BAX wild-type clones of OCI-Ly19 and Z-138 cells were effectively eliminated by apoptosis (sub-G1), the BAX-deficient clones of these sensitive lines showed a polyploid, G2M arrested population and no induction of cell death observed by both cell cycle analyses and by Annexin V/PI co-staining (Fig. 2c, d and Suppl. Fig. 3c–f).

Next, we explored the ability of BCL-2 and MCL-1 inhibitors to enhance PLK4 action in lymphoma. Venetoclax is a clinically approved BH3-mimetic drug that prevents BCL-2 binding to BAX, BAK, and other BH3-only proteins (BID, BAD, BIM, and PUMA)^46,56–58^. S63845 is an MCL-1 inhibitor that impairs the sequestration of a distinct but overlapping set of BH3 proteins (BAK, NOXA, BIM, and PUMA) by MCL-1^57–60^. We measured cytochrome c release in response to CFI, venetoclax, and S63845 treatment and observed that CFI effects were completely blocked in BAX-deficient cells, whereas venetoclax, and to a lesser extent S63845, retained some ability to trigger cytochrome c release indicating BAX-independent functions (Fig. 2e). Cell proliferation assays on OCI-Ly19 and Z-138 parental BAX-deficient clones confirmed BAX dependent effects of CFI and resistance to venetoclax, but also showed that the combination was able to completely overcome resistance to each and caused strong synergistic drug interactions (Fig. 2f and Suppl. Fig. 3g). Cell cycle analysis showed an accumulation of sub-G1/apoptotic cells and the elimination of the polyploid populations in BAX-deficient lines treated with the combination of CFI and venetoclax (Fig. 2g–i). Together, these results indicate that while CFI and S63845 depend solely on BAX, venetoclax can trigger BAX-independent cell death, most likely through BAK, and enable the elimination of PLK4-inhibited lymphoma cells.

### BCL-2 inhibition with venetoclax overcomes resistance to CFI-400945 in lymphoma

**Fig. 3:**
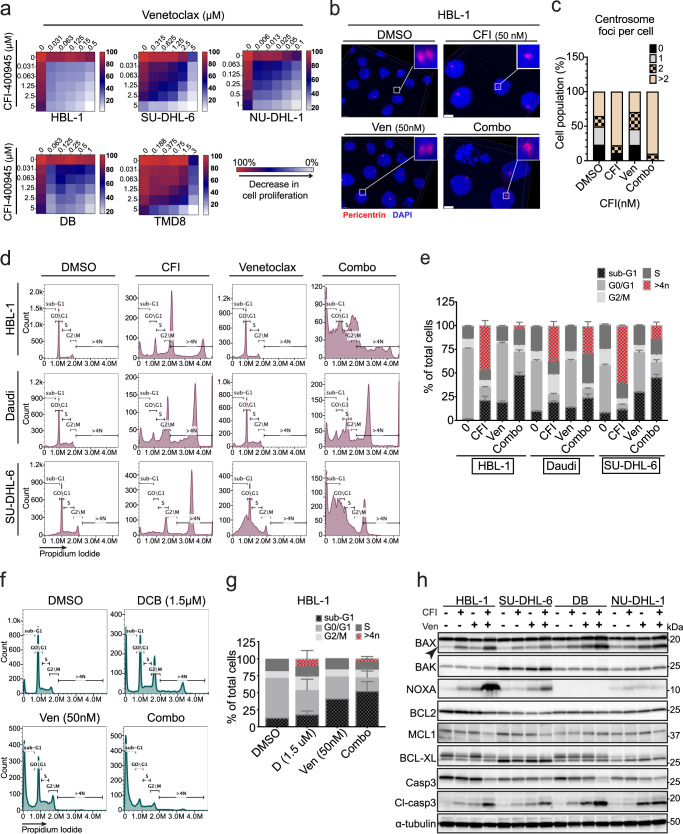
The BCL-2 inhibitor venetoclax synergizes with PLK4 inhibition and eliminates polyploid cells. **a** Heatmap representing the percentage of cell viability values for the CFI-resistant cell lines in response to CFI and venetoclax combined treatment for 72 h, as assessed by CellTiter-Glo assay. Cell viability values are represented in a heatmap colored from red (0% inhibition) or blue to white (50–100% inhibition). Values represent the average of three independent dose-titration curves (*n* = 3). **b** Representative confocal **c** quantification of OCI-Ly19 cells treated with CFI (25 nM), Venetoclax (25 nM), or a combination of both treatments for 72 h treatment. Cells were fixed and stained for pericentrin. Insets are 4X magnified. Scale bar, 3 μm. A minimum of three z-stack images were captured under the same condition. **d** Representative cell cycle analysis after PI staining of HBL-1, Daudi, and SU-DHL-6 cell lines after 72 h treatment of CFI (50 nM), venetoclax (50 nM), or combination (50 nM of each drug). **e** Quantification of cells at different stages of the cell cycle shows CFI-generated polyploid cells are eliminated in cell lines with high synergy between CFI and venetoclax. Data were presented as mean ± SD, *n* = 3. **f** Representative DNA histograms for cell cycle analysis of HBL-1 after 72 h treatment of Dihydrocytochalasin B (DCB) (1.5  μM), venetoclax (50 nM), or combination of both drugs. **g** Stacked bar graph showing the percentage of cells at various stages of the cell cycle in HBL-1 treated with DCB or venetoclax alone or with a combination of both drugs. Data were presented as mean ± SD, *n* = 2. **h** Western blot analysis of BCL-2 family proteins levels 72 h following combinational treatment with indicated drugs. Representative western blots from one of three experiments performed. α-tubulin is shown to indicate equivalence of loading. Ven Venetoclax, Combo combination of CFI and Venetoclax. Source data for panels **a**, **c**, **e**, **g**, **h** are provided as a Source Data file.

We then examined to what extent the combination of CFI and venetoclax would enhance killing across panels of CFI-resistant lymphoma lines. We quantified the degree of cooperation between the two drugs using the ΔBliss synergy values where negative values indicate synergy^61^ and a color-coded representation of cell killing where a concave/upward slope indicates a synergistic interaction (Fig. 3a and Suppl. Fig. 4a). The combination between CFI and venetoclax was strongly synergistic in the CFI-resistant lines HBL-1, NU-DHL-1, and SU-DHL-6, whereas Daudi, TMD8, SU-DHL-8 exhibited additivity in vitro (ΔBliss close to “0”) (Fig. 3a and Suppl. Figs. 4a, b). We confirmed that RNAi-mediated BCL-2 knockdown produced sensitization to CFI treatment (Suppl. Fig. 4c–f). Treatment with the MCL-1 inhibitor S63845 showed a distinct pattern, it was highly efficient in killing CFI-resistant cells (SU-DHL6, NU-DHL-1), less active against TMD8 and HBL-1 cells, but overall showed no synergistic drug effect with CFI (Suppl. Fig. 4g). In the CFI-resistant HBL-1 cells, venetoclax had no effect on centrosome number, CFI treatment increased the number of centrosome foci per cell, and the combination showed an increased number of disrupted centrosome structures (Fig. 3b, c). Cell cycle analysis of CFI-resistant HBL-1, SU-DHL-6, and Daudi cells, where CFI causes the accumulation of polyploid cells, showed that the combination with venetoclax largely eliminated these 4n cells and triggered massive cell death (Fig. 3d, e). We also extended our findings to another mechanism of drug-induced polyploidy with the inhibitor of actin polymerization, Dihydrocytochalasin B (DCB), that disrupts cytokinesis^62,63^. Briefly, DCB treatment resulted in approximately 20% tetraploid cells in HBL-1 cells that were readily eliminated by the addition of the BCL-2 inhibitor venetoclax (Fig. 3e–f). Hence, different mechanisms of drug-induced polyploidy synergize with BCL-2 inhibition in cell killing.

The cells showing the strongest drug synergy (e.g., HBL-1, SU-DHL-6, DB, and NU-DHL1) further showed an increase in BAX protein levels compared to lines that showed a less dramatic response (Fig. 3h and Suppl. Fig. 4h–k). A survey of the extent of BAX induction across all cells supports a P53-mediated increase by CFI as reported^30,31^, whereas the effect of the combination on BAX levels does not depend on wild-type P53 although at least in TMD8 cells, it is accompanied by an increase in the BAX mRNA (Suppl. Table 1). In this regard, we highlight a recent report that identifies a BCL-2-independent effect of venetoclax that triggers a metabolic and ATF4-mediated stress response^48^. Hence, the addition of venetoclax sensitizes CFI-resistant cells and triggers a robust cell death response that eliminates polyploid lymphoma cells.

### Non-malignant white blood cells are insensitive to CFI-400945 and venetoclax

**Fig. 4:**
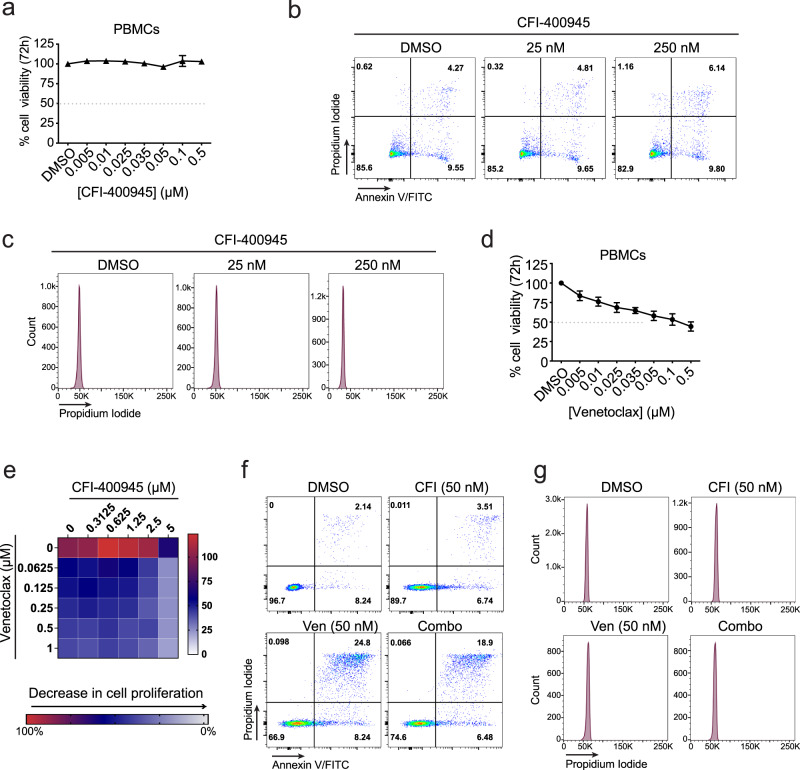
Effect of CFI and venetoclax combination on healthy PBMCs. **a** Cell viability of PBMCs isolated from whole blood from healthy donors measured by CellTiter-Glo after treatment with increasing concentrations of CFI compared to DMSO (0.1%) for 72 h. Values represent the mean ± SEM of 4 independent donors, each time in triplicate. **b** Representative flow cytometry analysis of PBMC apoptosis staining with Annexin V-FITC/Propidium Iodide (PI) after treatment with CFI (25 and 250 nM) or DMSO (0.1%). **c** Representative DNA histogram content- PI staining obtained by flow cytometry analysis in PBMCs treated with CFI (25 and 250 nM) or vehicle. **d** Cell viability of PBMCs (as shown in A) after treatment with venetoclax or DMSO (0.1%) for 72 h. Values represent the mean ± SEM of four independent donors, each time in triplicate. **e** Drug interaction matrix heatmap showing cell viability after 72 h treatment with CFI, venetoclax, or both. Cell viability values are represented in a heatmap colored from red (0% inhibition) or blue to white (50–100% inhibition). Heatmap grids correspond to the mean of three independent experiments/donors (*n* = 3). **f** Flow cytometric representation of apoptosis in PBMCs treated with DMSO (0.1%), CFI (50 nM), Venetoclax (50 nM), or both inhibitors for 72 h. Represented values are the mean ± SEM of two independent experiments. **g** Representative flow cytometry analysis of PBMCs stained with PI shows no alteration in the cell cycle in cells treated with either CFI and venetoclax alone or in combination. Ven Venetoclax, Combo combination of CFI and Venetoclax. Source data for panels **a**, **d**, **e** are provided as a Source Data file.

Next, we examined the effect of PLK4 inhibition with or without venetoclax on normal blood cells. Treatment of human blood mononucleated cells (PBMCs) across a relevant range of CFI concentrations (0–500 nM) had no effect on viability, indicating 200x-fold selectivity compared to sensitive OCI-Ly19 lymphomas (IC_50_ = 0.025 µM) (Fig. 4a). Flow cytometry analysis confirmed that CFI did not induce polyploidy or cell death in normal blood cells (Fig. 4b, c). Venetoclax induced cell death in primary PBMC samples with IC_50_ values between 50 and 100 nM (Fig. 4d). However, while CFI and venetoclax strongly synergize in lymphoma cells, this combination produces no increase in polyploidy or cell death induction in PBMCs (Fig. 4e–g). We further confirmed the same effects with the potentially more selective inhibitor centrinone (Suppl. Fig. 5a–c). Collectively, these in vitro data suggest a tumor-selective effect of CFI monotherapy and no increase in venetoclax toxicity in non-transformed B cells.

### In vivo efficacy and safety of the CFI-400945-venetoclax combination therapy

**Fig. 5:**
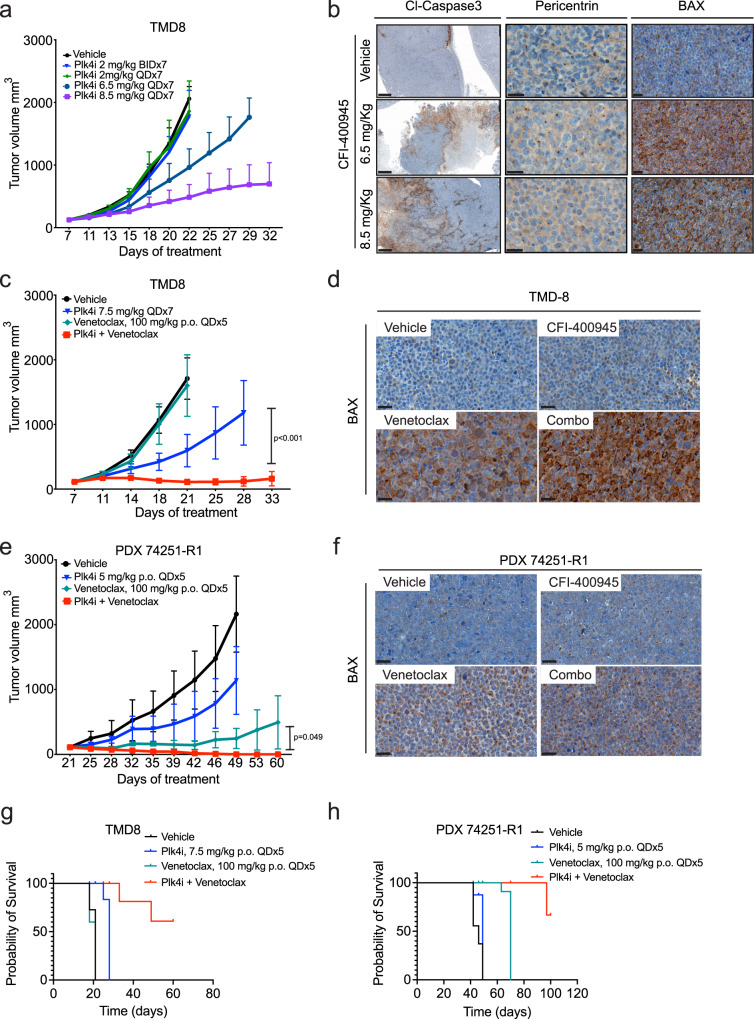
The combination of CFI and venetoclax is safe and highly effective against CFI-aggressive lymphomas in vivo. **a** NSG mice (*n* = 5 per group) bearing TMD8 xenograft tumors were treated with increasing doses of CFI (blue: 2 mg/kg QD; green: 2 mg/kg BID; dark blue: 6.5 mg/kg QD; purple: 8.5 mg/Kg QD; black: vehicle). Data represent the mean percentage ± SD (*n* = 5 animal/group). **b** Immunohistochemical analysis of cleaved (Cl) caspase 3, pericentrin, and BAX on CFI (6.5 or 8.5 mg/kg) and vehicle-treated TMD8 tumors. (20x magnification). At least three non-overlapping images were acquired for each condition. Scale bars: Cl-Caspase, 500 μm; Pericentrin, 20 μm; BAX, 30 μm. **c** TMD8 DLBCL xenograft model treated with CFI (Blue: 7.5 mg/kg, QD) and venetoclax (Green: 100 mg/kg QD) alone and in combination (red), vehicle control (black). Data were represented as mean percentage ± SD (*n* = 8 animals/group). **d** IHC staining for BAX on TMD8 xenografts shows a visible increase in BAX levels in venetoclax and combination-treated tumors. At least three non-overlapping images were acquired for each condition. Scale bar, 30 μm. **e** Genomically complex DLBCL PDX-74251-R1 with amplification of MYCL, mutations of *TP53*, RB1, KMT2D, CREBBP, TNFRSF14 (see text for details) (PDX-74251-R1) treated with CFI 5 mg/kg QD (blue) or 100 mg/kg Venetoclax (green) and the combination of same doses (red); Results represent mean ± SD of tumor volumes on different days (*n* = 6 animals/group. **f** Representative images of indicated IHC stains from PDX-74251-R1 tumors. At least three non-overlapping images were acquired for each condition. Scale bar, 30 μm. **g** Animal survival curves for mice harboring TMD8 xenografts or **h** PDX-74251-R following administration of CFI, Venetoclax, or in combination. [**h**, *n* = 8 animals/group; I, *n* = 6 animals/group)]. Additional safety toxicology data is available in the Suppl. Tables 3, 4 and Suppl. Data 1, 2. QD, once a day; BID, twice a day, p.o., oral administration. Statistics: *p* values were calculated by two-way ANOVA followed by Tukey’s multiple comparisons test. Source data for panels **a**, **c**, **e**, **g**, **h**, **i** are provided as a Source Data file.

We tested the efficacy and safety of the single agents and combination therapies in a cell line xenograft and a PDX model. Although these cells are relatively CFI resistant with an in vitro IC50 of 9.5 μM, these cells become significantly more sensitive with the addition of venetoclax (Fig. 3a). Subcutaneous xenografts of TMD8 cells engrafted well, and we initiated treatment when tumors reached a size of 100 mm^2^. A low-dose regimen of CFI (2 mg/Kg QDx7 or 2 mg/Kg BIDx7 for 3 weeks) had no effect. Higher CFI doses (6.5 mg/Kg QDx7 and 8.5 mg/Kg QDx7, for 3 weeks) induced partial responses (*p* = 0.01 and *p* = 0.0007, respectively, *n* = 5 animals per group) (Fig. 5a). The high CFI doses produced defects in centrosome number, BAX accumulation, and caspase 3 cleavage, indicating apoptosis when compared with the control group (Fig. 5b and Suppl. Figs. 6a, b). Single-agent venetoclax (100 mg/kg, p.o., QDx5) had no anti-tumor effect in TMD8 xenograft (Fig. 5c). However, the combination of CFI (7.5 mg/Kg QDx7, 3 weeks) with venetoclax (100 mg/Kg QDx5, 3 weeks) produced nearly complete responses (*p* < 0.0001) (Fig. 5c), which was associated with increased BAX protein levels and caspase 3 cleavage in tumors treated with the combination (Fig. 5d and Suppl. Fig. 6c, e, f). Additionally, tumors treated with CFI exhibited centrosome overduplication and this phenotype was attenuated with the combination with Venetoclax (Suppl. Fig. 6c, g).

We then tested the combination of PLK4 and BCL-2 inhibitors in a primary transplanted patient-derived GCB-DLBCL lymphoma model (PDX). The mutational profiling indicated a complex genotype (Suppl. Table 2) which might facilitate the survival of aneuploid tumor cells. In detail, the PDX tumor harbors a genomic amplification of the *MYC* gene, missense mutations in *TP53* (R158H), and a splice mutation in the *RB1* gene, along with mutations in the genes encoding the histone H3K4^me1/2^ methyl transferase *KMT2D* (Q3601*)^64^, the histone acetyltransferase *CREBBP* (Q935Hfs*63)^65^, and genomic loss of the gene for the tumor suppressive receptor *TNFRSF14/HVEM*^66^. Treatment with CFI alone (5 mg/kg, QDx5) had a partial effect on these complex PDX tumors. Venetoclax (100 mg/Kg QDx5, *p* < 0.0001) produced partial responses. However, the combination of CFI and venetoclax showed synergy and caused complete and lasting responses in this complex tumor such that no visible tumors were detected up to 42 days following the administration of the first dose (*p* < 0.0001, *n* = 15) (Fig. 5e).

Tumor histology confirmed tumor cell death with increased caspase 3 cleavage and BAX levels (Fig. 5f and Suppl. Fig. 6d–f) as well as the elimination of abnormal pericentrin levels and cells with multiple centrosomes in animals treated with the combination therapy (Suppl. Fig. 6d–g).

CFI and venetoclax combination did not result in increased toxicity in vivo, as treatment did not cause mortality or weight loss in mice (Suppl. Fig. 6h–j). Notably, the same combination significantly increased the survival of mice bearing either TMD8 xenografts or PDX tumors compared to animals receiving each drug alone (*p* < 005; *n* = 6–8 animals per treatment cohort) (Fig. 5g, h). Furthermore, treatment did not induce damage to vital organs (Suppl. Fig. 6k) and was not associated with any abnormalities in liver, kidney, or bone marrow functions. (Suppl. Tables 3, 4 and Suppl. Data 1, 2).

## Discussion

Our results reveal drug-induced polyploidy as a therapeutic vulnerability in lymphoma. Aneuploidy and polyploidy are rare events in B cell lymphomas. Accordingly, adaptive mutations in genome integrity checkpoints that enable the survival of genomically unstable cells are rarely seen in human lymphomas compared to solid cancers^1,13,14,30^. Inhibition of PLK4 acutely causes genomic instability, and in the absence of adaptive mutations, polyploid lymphoma cells are rapidly eliminated via P53^30,31^ and BAX^67^-mediated apoptosis. Accordingly, BAX-deficient lymphomas are highly resistant to the PLK4 inhibitor CFI-400945. Inhibition of BCL-2 with venetoclax releases BAX and BAK from BCL-2 and this enables the killing of PLK4-inhibited cells even in the absence of BAX and most likely through BAK^46,68^. The combination of CFI and venetoclax further shows additive and synergistic effects across panels of lymphoma cell lines and in xenograft and PDX models in vivo. A surprising observation is that combined venetoclax and CFI increases BAX levels in an apparently P53-independent manner and in cells and tumors where CFI alone does not increase BAX expression. This finding is not readily explained by simple disruption of BAX-BCL-2 interaction and suggests that venetoclax has activities beyond BCL-2 and is potentially consistent with reported BCL-2 independent venetoclax effects on cell metabolism that cause activation of the ATF4 cell stress response program^48^.

We chose to study the PLK4 inhibitor CFI-400945 because it is the most potent inhibitor and is currently in clinical trials against a wide range of solid tumors. Despite a large amount of prior work in other cancer models^27,28,31,32,36,39,55^, questions remain as to the selectivity of CFI for PLK4 over other kinases and especially Aurora B^4,28,37,38^. Manson et al.^39^ documented 100-fold selectivity for PLK4 compared to Aurora B. We find CFI causes PLK4 inhibition, polyploidy, and cell death at 10-25 nM, whereas we begin to detect evidence of Aurora B inhibition at 100 nM. Consistently, we observed few multinucleated cells that would be expected with Aurora B inhibition, and we further replicated key findings with the, even more, PLK4 selective centrinone compound^37^. However, it is still possible that some Aurora B inhibition occurs, particularly in vivo, which might result in cytokinesis failure and would further increase polyploidy that contributes to the observed anti-lymphoma effects^38,69,70^. Our findings extend beyond PLK4 inhibition with either CFI or centrinone, and we made analogous observations with Cytochalasin D, an inhibitor of actin polymerization that also causes accumulation of polyploid cells that are rapidly eliminated upon BCL-2 inhibition.

Venetoclax and the PLK4 inhibitor CFI-400945 are already in clinical use, and our data provide a mechanistic rationale for testing their combination in the treatment of aggressive lymphomas. Specifically, venetoclax is approved for the treatment of chronic lymphocytic leukemia and small lymphocytic lymphoma and shows variable single-agent efficacy^45,71^. CFI is in clinical trials against a range of malignancies and not including lymphoma (NCT03187288, NCT04176848, NCT01954316, NCT04730258, NCT03624543, and NCT03385655). Importantly, while increased and cumulative toxicity is a major concern, for example, in combinations of venetoclax and doxorubicin^31^, we find the combination of PLK4 inhibition with venetoclax is well tolerated. This reflects both the selectivity of venetoclax for BCL-2 and the PLK4 indifference of non-proliferating tissues. Moreover, a recent Phase 1 study did observe neutropenia as a dose-limiting CFI toxicity^55^, and consistently our own data show only a modest reduction in blood counts. Together, we report a synergistic drug combination based on the biological properties of lymphoma, which are ill-prepared for polyploidy and susceptible to combined PLK4 and BCL-2 inhibition to induce and eliminate polyploid cells with negligible toxicity in healthy animals.

## Methods

### Relevant ethical regulations

All animal studies were reviewed and approved by the Institutional Animal Care and Use Committee (IACUC) at Jubilant BioSys, Memorial Sloan Kettering Cancer Center (MSKCC), and START, Center for Cancer Care. All mice were maintained in accordance with the guidelines of the Association for Assessment and Accreditation of Laboratory Animal Care International (AAALAC) on the care, welfare, and treatment of laboratory animals, and all experiments were conducted under approved protocols from Institutional Animal Care and Use Committees (IACUC).

### Cell culture

The human mantle cell lymphoma (MCL) cell lines (REC-1, JeKo-1, Z-138, Mino, JVM-2, JVM-13), Burkitt lymphoma (BL) cell lines (Raji, EB1, Daudi, Ramos, and CA46) and solid tumors cell lines were obtained from ATCC (American Type Culture Collection). Diffuse large B cell lymphoma (DLBCL)-derived cell lines (SU-DHL-4, SU-DHL-6, SU-DHL-8, SU-DHL-10 OCI-Ly19, DB, NU-DHL-1, U-2973, OCI-Ly-3, U-2932, RI-1, and OCI-Ly-10), Hodgkin’s cell lines (L-428, HDLM-2, and KM-H2), and the MCL cell line MAVER-1 were obtained from the DSMZ-German Collection of Microorganisms and Cell Cultures, Department of Human and Animal Cell Cultures (Braunschweig, Germany). The cell lines BJAB (BL), HBL-1 and TMD8 (DLBCL), SUP-M2, SU-DHL-1, and KARPAS-299 (Anaplastic Large Cell Lymphoma (ALCL)-cell lines) were provided by Dr. R.E. Davis (MD Anderson Cancer Center, Houston, TX). Lymphoma cell lines were authenticated by STR analysis at the Integrated Genomic Operation Core Facility at Memorial Sloan Kettering Cancer Center, New York, NY. Cells were cultured and maintained in RPMI 1640 medium supplemented with 10–20% heat-inactivated fetal bovine serum (Hyclone, GE Healthcare Life Sciences), 1% l-glutamine, and penicillin-streptomycin in a humid environment of 5% CO_2_ at 37 °C. Cells were routinely tested and confirmed negative for mycoplasma in-house using the mycoplasma detection kit available from Lonza Biosciences (cat.#LT07-218) according to the manufacturer’s instructions. Mutations were annotated according to Cancer Cell Line Encyclopedia (https://www.broadinstitute.org/ccle/home).

### Western blotting

Preparation of cellular protein lysates or tumor samples was performed by using the Cell Signaling lysis buffer (#9803) according to the manufacturer’s extraction protocol. Protein quantitation was done using the Direct Detect system (Millipore) or Coomassie Blue reagent following the recommended protocol. A total of 30 μg of protein was denatured in Laemmli buffer supplemented with DTT (10%) at 95 °C for 5 min. Western immunoblotting was performed using the Bio-Rad system (TGX 4–15% gels) or Expressplus PAGE 4–12% gel was assembled in an XCell II Blot Module (Invitrogen, Catalog no. EI9051). The transfer was performed for 7 min using the Trans-Blot turbo system (Bio-Rad) onto PVDF or nitrocellulose membranes. The blots were incubated overnight in 10 mL of blocking buffer (5% BSA in PBS) containing primary antibody. Secondary antibody incubation was done in a blocking buffer containing HRP-conjugate secondary antibody for 1 h at room temperature and protected from light. Images were acquired by using the Bio-Rad Imaging Chemidoc MP system using the Bio-Rad Image Lab 6.1 Software for Mac.

The following antibodies for western blotting were purchased from Abcam: NOXA (cat. ab13654, dilution 1:1000), STIL (ab89314, dilution 1:1000) and pericentrin (ab4448, dilution, 1:250). BAX (#2774 S, dilution, 1:1000), BAK (#12105 S, dilution, 1:1000), Cleaved Caspase 3 (#9661, dilution, 1:1000), Caspase 3 (#9662, dilution, 1:1000), BCL-2 (#4223 S, dilution, 1:1000), BCL-xL (#2764 S, dilution, 1:1000), MCL-1 (#4572 S, dilution, 1:1000), PUMA (#12450 S, dilution, 1:1000), alpha-tubulin (# 3873, dilution, 1:10000) were purchased from Cell Signaling. beta-Actin (#5316, dilution 1:10000) was purchased from Sigma, CEP110 was purchased from EMD Millipore (MABT1354, dilution 1:250) and PLK4 (#12952-1-AP, dilution, 1:1000) was purchased from Proteintech. Biotinylated rabbit anti-goat lgG (Cat# BA-5000, dilution, 1:10000) and Biotinylated goat anti-rabbit lgG (Cat# PK-6101, dilution, 1:10000) were supplied by Vector Labs.

### CRISPR Knockout

Z-138 and OCI-Ly19 cells were transduced with lentiCas9-Blast from Addgene (Cambridge, MA). Forty-eight hours after transduction, cells were selected using 10–20 μg/ml blasticidin for 5 days and sorted. Cas9 stable cell lines were transduced with puromycin resistance cassette from LentiGuide-Puro vector backbone (Addgene 52963) with the following guide RNA sequences: BAX KO1: GGACGAACTGGACAGTAACA; BAX KO2: GTTTCATCCAGGATCGAGCA. Z-138 and OCI-LY19 cells were transduced with lentiviral sgRNAs particles and 3 days post-transduction GFP-positive cells were sorted. Successful knockout was confirmed by immunoblotting.

### Cell proliferation assay and combination assay

Suspension cells were seeded in 96-well plates at 25,000 cells per 100 μL per well or in 24-well plates at 250,000 cells per 1 mL per well with either vehicle (DMSO 0.1%) or increasing concentrations of the compound for 24, 48, and 72 h. Cell viability was assessed with CellTiter-Glo Luminescent assay (Promega). About 80 μL of the cultured cells were transferred to opaque-white bottom 96-well plates and mixed with 20 μL of CellTiter-Glo reagent. The mixture was incubated for 30 min at RT and read using an EnSight Multimode plate reader (PerkinElmer) with Kaleido version 1.2. to collect luminescence. Data were normalized as percentage viability and graphed by nonlinear regression curves using GraphPad Prism® 7 software.

To establish the interaction between two drugs in combination assays, we calculate the log-odds; the value: log base 2 (observed growth inhibition/product of the two fractional growth inhibitions). A log-odds value of zero indicates that the combination treatments are additive; a negative value indicates synergy between the drugs and a positive value indicates antagonism.

### Cell cycle and apoptosis analysis by Flow cytometry

Cell lines were cultured and treated with DMSO or CFI for 72 h. Cells were collected and then stained with propidium iodide (PI)/RNase staining solution (Cell Signaling, #4087) at room temperature for 15 min. Flow cytometric data were acquired on a BD FACSCalibur (BD Biosciences, San Jose, CA) using CellQuest Pro Version 6.0. Propidium iodide was excited by the 488 nm laser and fluorescence emission was measured in fluorescence parameter 3 (FL3)—with the standard 670LP filter. Greater than 10,000 events were acquired. Data were analyzed with Flowjo software. Doublets were excluded by gating out high FL3- W (width) cells.

Apoptotic cells were quantified using the FITC Annexin V Apoptosis Detection Kit (BD Pharmigen, #556547. Cells were seeded at a density of 5 × 10^5^ cells /ml in six-well plates. After treatment, cells were harvested, and labeling was performed according to the manufacturer’s instructions. Stained cells were analyzed using the Aurora or Fortessa cytometer. Both Annexin V- and PI-negative cells [annexin V−/PI−; quadrant 3] were considered normal survival cells; annexin V-positive and PI-negative and (annexin V+/PI−; quadrant 4) cells were thought to be in the early apoptotic stage; annexin V-and PI-positive (annexin V+/PI+; quadrant 2) cells were thought to be in the late apoptotic stage; annexin V-negative and PI-positive (annexin V−/PI+; quadrant 1) cells were considered mechanically injured cells. GFP-positive cells were analyzed using the PE Annexin V Apoptosis Detection Kit (BD Pharmigen, #559763).

### Real-time PCR assay

Total RNA was extracted using AllPrep DNA/RNA/Protein Mini Kit (Qiagen, Hilden, Germany, 80004). cDNAs were synthesized from 1 µg of total RNA using SuperScript III First-Strand (Invitrogen, Waltham, MA, USA, 18080-400) and were amplified using TaqMan Universal Master Mix II, no UNG (Applied Biosystems, Waltham, MA, USA, 4427788). BAX, the forward primer is 5′-TCTGAGCAGATCATGAAGACAG−3′ and the reverse primer is 5′-CCACTCGGAAAAAGACCTCT-3′, this corresponds to IDT primetime assay ID: Hs.PT.56a.19141193.g. Analysis was performed by ∆∆Ct. Relative mRNA expression was evaluated after normalization for GAPDH expression. Data show results from three independent experiments.

### Immunofluorescence and quantification of centrosome

Lymphoma cells were washed with PBS and placed on coated glass coverslips (Shi-fix^TM^, #SB-Shifix25) for 30 min for adhesion, fixed in 4% PFA in PBS for 15 min at room temperature, and immunostained for pericentrin (Abcam, ab4448, dilution, 1:250), CEP110 (Millipore, MABT1354, dilution, 1:250) and nucleus was stained with DAPI (Molecular Probes, #D1306, dilution, 1:10000). Permeabilization was performed with PBS with 0.5% Triton X-100 for 30 min and blocking with 1% BSA in PBS for 1 h. Confocal z-stacks were taken on the LSM880 scanning confocal microscope (Zeiss, Germany) with Airyscan using a 63x/1.4NA objective. The images were then processed with Airyscan processing and converted into.ims files to create 3D images using Imaris 10.0 (BITPLANE, Switzerland). For quantification, DAPI was segmented in ImageJ v1.54 and the 3dMaximaFinder plugin was used to detect and count the individual centrosomes within each nucleus based on their relative maximum intensity.

### Detection of cytochrome c release

OCI-Ly19 isogenic cell lines were pre-treated with different compounds (CFI-400945, venetoclax, S63845, or vehicle (DMSO)) for 48 h at 37 °C. Cytochrome c release was measured using a plate-based in the iBH3 profiling assay previously described in ref. ^72^. About 2 × 10^5^ cells/ml were resuspended in MEB buffer (150 nM Mannitol, 10 mM HEPES, 150 mM potassium chloride, 1 mM EGTA, 1 mM EDTA, 0.1% BSA, 5 mM Succinate, and 2.5 g/L of Polaxamer 188 in distilled water at pH 7.5). Cell suspension (50 µL) was added to 50 µL of MEB Buffer containing 0.002% digitonin for 60 min at 25 ±3 °C. After incubation, cells were fixed with 4% formaldehyde for 10 min and then neutralized with N2 buffer (1.7 M Tris base, 1.25 M Glycine, pH 9.1). Cells were stained with anti-cytochrome C antibodies to quantify the remaining intracellular cytochrome c. After incubation, stained cells were analyzed on a BD LSRFortessa cell analyzer and FlowJo, LCC, 10.8 was used for posterior data analysis. The percentage of mitochondrial depolarization was calculated using by the normalized median fluorescence intensity (MFI) using dimethyl sulfoxide (DMSO) as negative release control and Alamethicin as positive release control (100% depolarization). The following equation was used to calculate the percentage depolarization for each peptide: %Cytochrome c loss = 1 − [(MFI_peptide/sample_) − (MFI_Pos Ctrl_)]/[(MFI_Neg Ctrl_) − (MFI_Pos Ctrl_)].

### Animal studies

Female NSG mice (NOD scid gamma mice) were obtained from the Jackson laboratory. Six-week-old NSG female mice were injected subcutaneously with 10 million tumor cells together with matrigel. Once tumors reached a volume of 100 mm^3^, mice were randomized to receive either vehicle control (0.5% methylcellulose) or specified single agents or combinations. Mice were observed daily throughout the treatment period for signs of morbidity/mortality and body weights were recorded twice a week. Tumors were measured two to three times per week using calipers, and volumes were calculated using the formula: length × width^2^ × 0.52. Mice were sacrificed by CO_2_ asphyxiation followed by cervical dislocation once the tumor volume exceeded 2000 mm^3^ or body weight loss was higher than 10%. The analysis of animal survival data is based on pre-terminal disease and/or a large tumor burden necessitating euthanasia. Upon completion of the experiment, tumors were harvested and processed for immunoblotting or immunohistochemistry assays.

### Immunohistochemistry

IHC analysis was performed at the Molecular Cytology Core Facility of Memorial Sloan Kettering Cancer Center using a Discovery XT processor (Ventana Medical Systems). Three hours after the last drug treatment, tumors were immediately fixed in fresh 4% paraformaldehyde rotating at 4 °C overnight. Fixed tissues were dehydrated and embedded in paraffin before 5 µm sections were put on slides. The tissue sections were deparaffinized with EZPrep buffer (Ventana Medical Systems) and incubated with antibody against stated antibodies for 5 h, followed by 60 min of incubation with biotinylated mouse secondary (Vector Labs, cat. BMK-2202, MOM in 5.75 ug/mL (1:200)). Slides were scanned with Pannoramic 250 Flash scanner (3DHistech, Hungary) using 20X (0.8 NA) objective lens. Tunel assay for mouse organs was performed following standard protocols at Histowiz, Inc (Brooklyn, NY).

### Statistics

Specific statistical parameters and analysis are stated in corresponding figure legends for each panel of data. Analyses were carried out within the programs GraphPad Prism 9.5.0 and the R statistical environment. Tests resulting in *p* values less than 0.05 were deemed significant. Error bars reflect the standard error of the mean (SEM) or standard deviations (SD) as stated in the respective legends. Boxes in whisker plots extend from the 25th to 75th percentiles and the middle line is plotted at the median, with whiskers to maxima and minima of all data. Group comparisons were tested by two-way ANOVA followed by Tukey–Kramer’s honestly significant difference test. Differences were considered statistically significant at *p* < 0.05. For synergy estimation, ΔBliss excess was calculated, as shown previously (Wilson et al., 2014). For animal experiments, three biological specimens we analyzed for each condition. In vitro experiments were performed in triplicate and repeated at least three times. In all experiments, attempts to replicate the experiments were successful. At least three independent sets of western blot and were performed to ensure reproducibility, and representative data were shown. For quantification of centrosome markers’ immunofluorescence and immunohistochemistry in tumor sections, three independent experiments or subjects, respectively, were analyzed unless stated otherwise, and representative data were shown. All imaging data acquisitions for quantitated analyses and their quantitation were performed with the researcher blind to the conditions.

### Reporting summary

Further information on research design is available in the Nature Portfolio Reporting Summary linked to this article.

## Supplementary information

Supplementary information

Reporting Summary

Description of Additional Supplementary Files

Supplementary Data 1

Supplementary Data 2

## Data Availability

The datasets generated during and/or analyzed during the current study are available and provided within the Article, Supplementary information, or Source Data file. Source data are provided with this paper.

## Source data

Source Data file
