# Supplementary material for: Synthetic lethality of drug-induced polyploidy and BCL-2 inhibition in lymphoma
Source: Nat Commun. 2023 Mar 18;14:1522. doi: 10.1038/s41467-023-37216-2 (PMC10024740; doi:10.1038/s41467-023-37216-2)
Supplement: Supplementary file 1 — Supplementary information [file 41467_2023_37216_MOESM1_ESM.pdf]

Supplementary information:

Synthetic lethality of drug-induced polyploidy and BCL-2 inhibition in lymphoma

Ana Portelinha[1,2], Mariana da Silva Ferreira[1], Tatiana Erazo[2], Man Jiang[1], Zahra Asgari[2], Elisa de Stanchina[3], Anas Younes[2,*], Hans-Guido Wendel[1*]

[1]Cancer Biology & Genetics Program, Memorial Sloan-Kettering Cancer Center, New York, NY 10065, USA.

[2]Department of Medicine Lymphoma Service Memorial Sloan-Kettering Cancer Center, New York, NY 10065, USA.

[3]Antitumor Assessment Core, Memorial Sloan Kettering Cancer Center, New York, NY, USA.

* Corresponding Authors: (Lead contact) Hans-Guido Wendel: Wendelg@mskcc.org

Anas Younes: anas.younes@astrazeneca.com

Supplementary figures

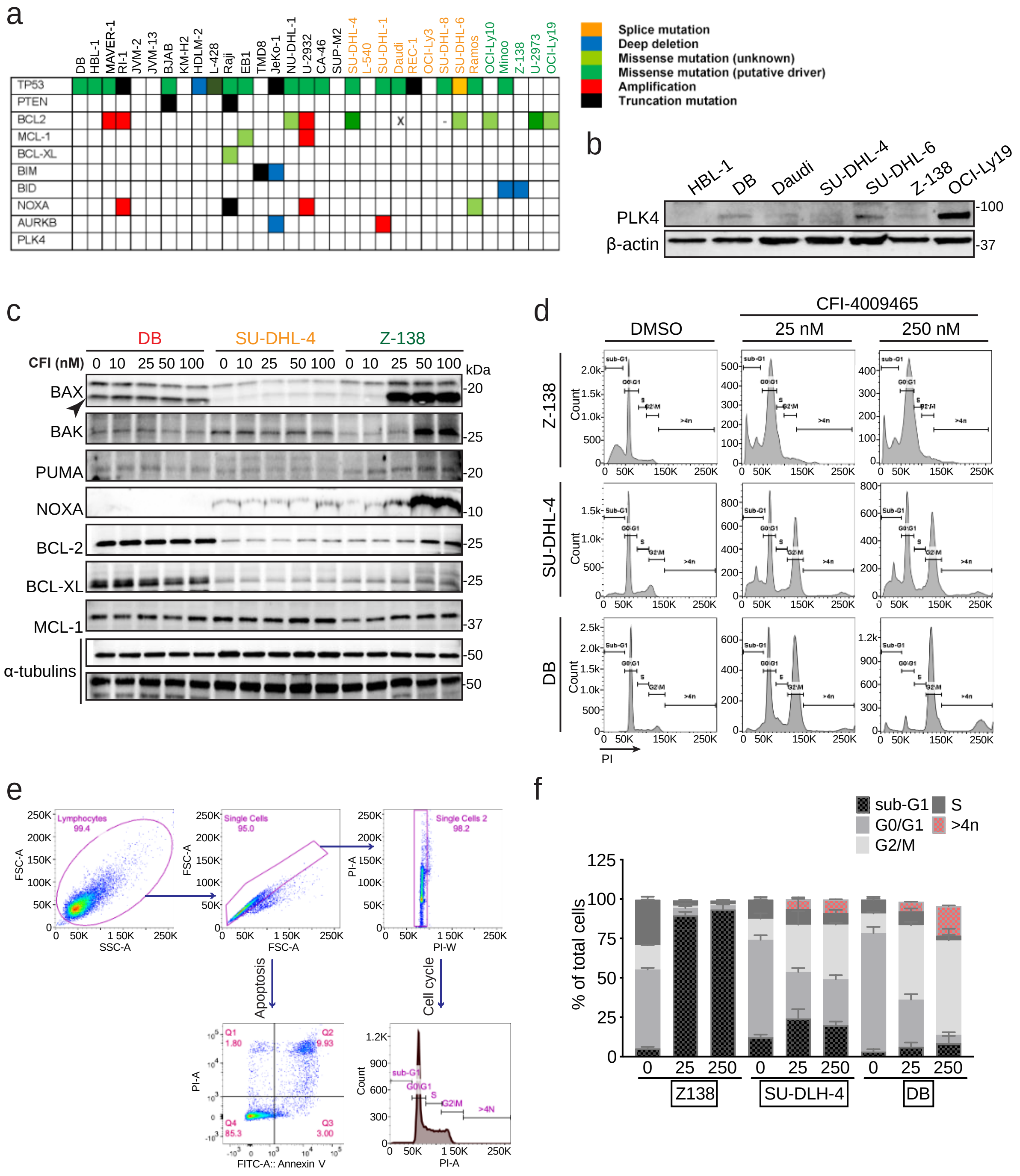

Supplementary figure 1: CFI activity in a panel of lymphoma cell lines. a) Mutational map of lymphoma cell lines. The panel includes genes involved in apoptosis, cell cycle and proliferation.

Each row in the figure corresponds to one gene in cBioPortal library, whereas each column corresponds to a designated cell line. **b)** Western blot showing basal expression of PLK4 in different lymphoma cell lines. PLK4 expression levels do not correlate with response to CFI therapy. β-actin indicates equivalence of loading. **c)** Western blot analysis of anti- and pro-apoptotic Bcl-2 family proteins in CFI resistant (DB), intermediate (SU-DHL-4) and sensitive (Z-138) cell lines treated with increasing concentrations of CFI for 72h. Sensitive and cell lines exhibit overexpression of pro-apoptotic proteins after treatment with CFI at the indicated doses. α-tubulin indicates equivalence of loading. Each immunoblot result on panel **b** and **c** is the representative of at least three repeats**. d)** Representative flow cytometric analyses of cell cycle distribution by propidium iodide (PI) staining in CFI sensitive (Z-138), intermediate (SU-DHL-4), and resistant (DB) cell lines 72 h after CFI treatment (25 or 250 nM) or control (DMSO 0.1%). Data is representative of two independent experiments. **e)** Flow cytometry-based gating strategy used for cell cycle analysis and apoptosis analysis. Total cells are gated to exclude cellular debris plotting SSC-A *vs*. FCS-H and doublets were discriminated by single (apoptosis) or double (cell cycle) exclusion by plotting FSC-A *vs*. FSC-H following plotting PI-A *vs*. PI-W. The first peak on cell cycle histogram corresponds to G1 cycle cells, whereas the second peak to the G2 cycle cells, with cells in phase S in between. To measure apoptosis, different cell populations were selected and plotted onto an AnnexinV FITC against a propidium iodide dot plot to access the levels of apoptosis. Early apoptotic cells positive for annexin V can be seen in the bottom right quadrant and(Q3) and late apoptotic cells positive for both annexin and PI in the top right quadrant (Q2). Necrotic cells positive for PI only are seen in quadrant 1 (Q1). Healthy cells are negative for both stains (Q4). **f)** Stacked bar graph representing the cell-cycle distribution of PI staining in three lymphoma cell lines treated as in panel *d*. CFI induced a specific G2/M arrest and polyploidy in the resistant cell lines and an increase in sub-G1 population on the CFI-sensitive cell lines. Data represent mean ± SD of duplicate experiments. Source data for panels **b** and **c**, are provided as a Source Data file.

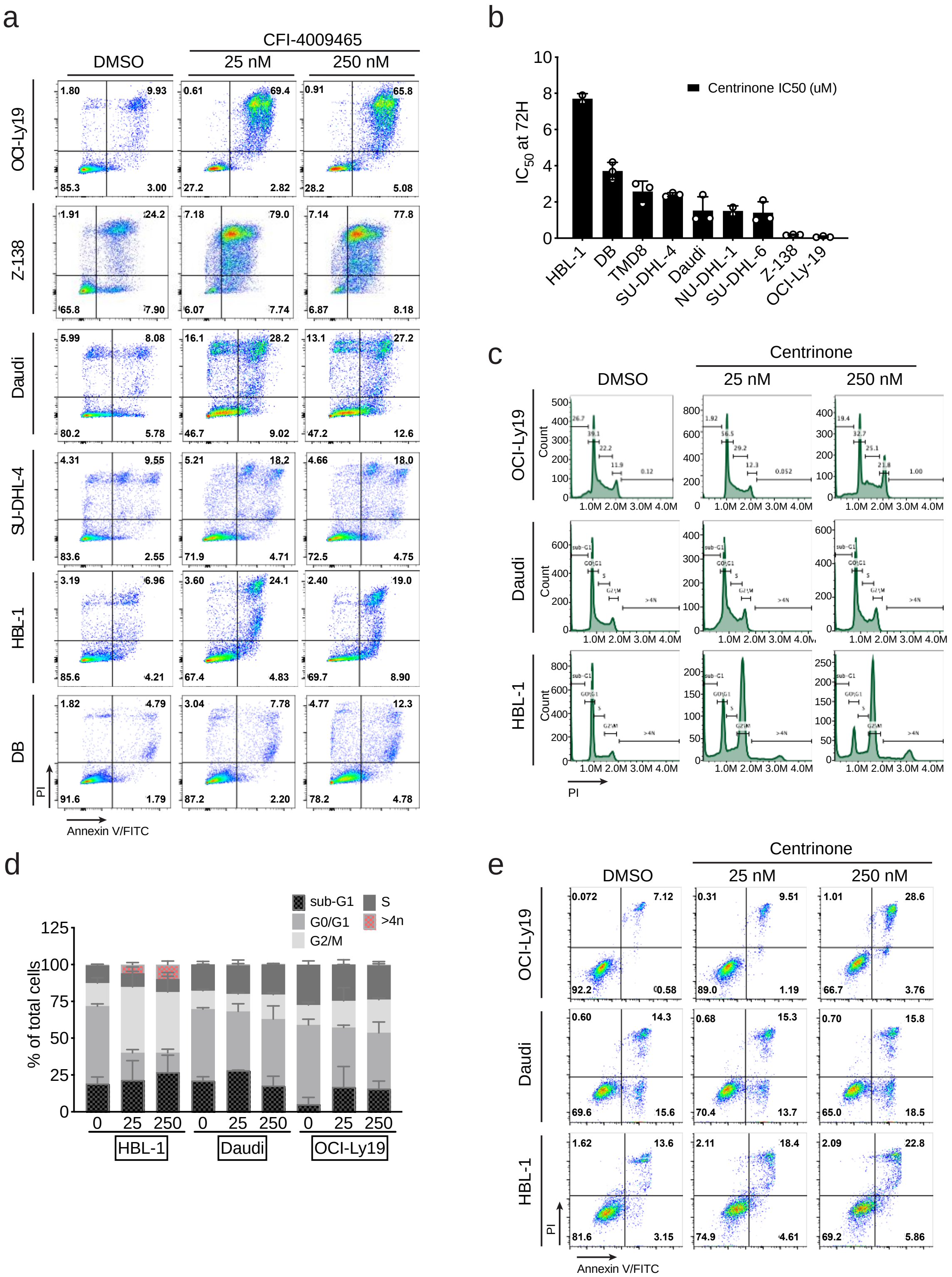
a
CFI-4009465
DMSO
25 nM
250 nM
OCI-Ly19
Z-138
Daudi
SU-DHL-4
HBL-1
DB
PI
Annexin V/FITC
b
Centrinone IC50 (uM)
IC50 at 72H
HBL-1
DB
TMD8
SU-DHL-4
Daudi
NU-DHL-1
SU-DHL-6
Z-138
OCI-Ly-19
c
Centrinone
DMSO
25 nM
250 nM
OCI-Ly19
Daudi
HBL-1
Count
PI
d
sub-G1
S
G0/G1
>4n
G2/M
% of total cells
HBL-1
Daudi
OCI-Ly19
e
Centrinone
DMSO
25 nM
250 nM
OCI-Ly19
Daudi
HBL-1
PI
Annexin V/FITC

Supplementary figure 2: CFI and centrinone activity in lymphoma cell lines. a) Representative dot plots of Annexin V-FITC/propidium iodide (PI) staining in CFI sensitive (OCI-Ly19, Z-138), intermediate (Daudi, SU-DHL-4), and resistant (HBL-1, DB) cell lines 72 h after CFI treatment (25 or 250 nM) or control (DMSO 0.1%) show significant increase of apoptotic levels in CFI-sensitive after 72h of treatment (25 and 250nM). **b)** Centrinone IC_{50} values in a panel of cell lines representing different sub-types of lymphoma treated for 72h. Cell viability was measured by CellTiter-Glow assay. Each bar represents the mean ± SD of 3 independent experiments, each time in triplicate. **c)** Representative flow cytometric analyses of cell cycle distribution by propidium iodide (PI) staining in OCI-Ly19, Daudi, and HBL-1 cell lines 72 h after centrinone treatment (25 or 250 nM) or control (DMSO 0.1%). Centrinone induces polyploidy in HBL-1 cells treated with 250 nM of centrinone **d)** Stacked bar graph representing the cell-cycle distribution of PI staining in three lymphoma cell lines treated with either control (DMSO, 0.1%) or centrinone (25nM or 250 nM) for 72h. Values represent mean ± SD of duplicate experiments. **e)** Representative dot plot of the Annexin V-FITC/PI assay showing apoptotic levels of lymphoma cells treated with centrinone (25 and 250nM) or DMSO (0.1%). Source data for panels **a** and **c** are provided as a Source Data file.

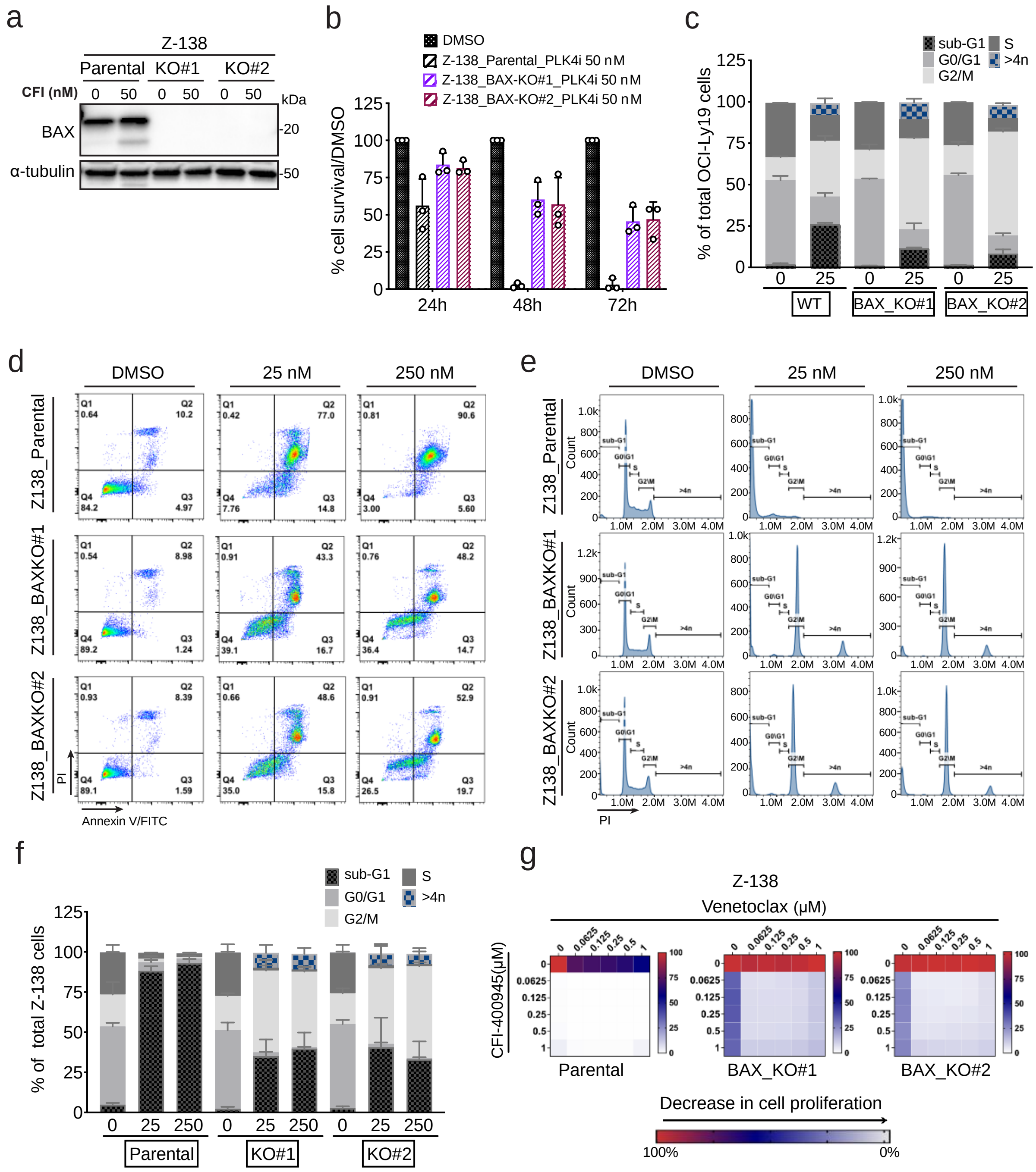

Supplementary Figure 3: BAX-dependent response to CFI treatment, part 2. a) Confirmation of BAX protein depletion by Western Blot in Z-138 cells and isogenic Z-138 BAX knockout (KO) clones (BAXKO#1 and BAXKO#2) treated with CFI (50 nM) or with control (DMSO, 0.1%). Immunoblot result is the representative of three repeats. **b)** Cell proliferation of Z-138 and isogenic

clones after 24, 48 and 72 h of treatment with 50nM of CFI or with DMSO. Cell viability was assessed by using CellTiter-Glo luminescent assay and normalized to DMSO-treated cells at each time point. The data is expressed as the mean ± SD of three independent experiments in triplicates. **c)** Stacked bar graphs showing the percentage of cells at various stages of cell cycle (SubG1/Apoptotic, G0/G1, S, G2/M and >4n) in OCI-Ly19 WT and OCI-Ly19 BAX-knockout cells after incubation with 25 nM of CFI or DMSO (0.1%) for 72h. The results are expressed as the mean ± SD of two independent experiments. **d)** Representative dot plot of the Annexin V-PE/propidium iodide (PI) assay showed less apoptotic induction in Z-138 BAX-knockout cells treated with 25 nM for 72h compared to the Z-138 BAX-WT cells. **e)** Flow cytometry analysis on cell cycle progression using PI staining in different lymphoma Z-138 BAX knockout and WT cell lines upon 72 h of CFI (25 or 250 nM) or control (DMSO 0.1%) treatment. **f)** Stacked bar graphs showing the percentage of cells at various stages of cell cycle (SubG1/Apoptotic, G0/G1, S, G2/M and >4n) in Z-138 isogenic cell lines after incubation with 25 nM of CFI or DMSO (0.1%) for 72h. The data is expressed as the mean ± SD of three independent experiments (n=3). **g)** Heat map representing percentage of cell viability values for the Z-138 isogenic cell lines in response to CFI and venetoclax combined treatment for 72h, as assessed by CellTiter-Glo assay. Cell viability values are represented as in a heatmap colored from red (0% inhibition) or blue (50% inhibition) to white (100% inhibition) and were the average of 2 independent experiments. Source data for panels **a**, **b**, **c**, **f** and **g** are provided as a Source Data file.

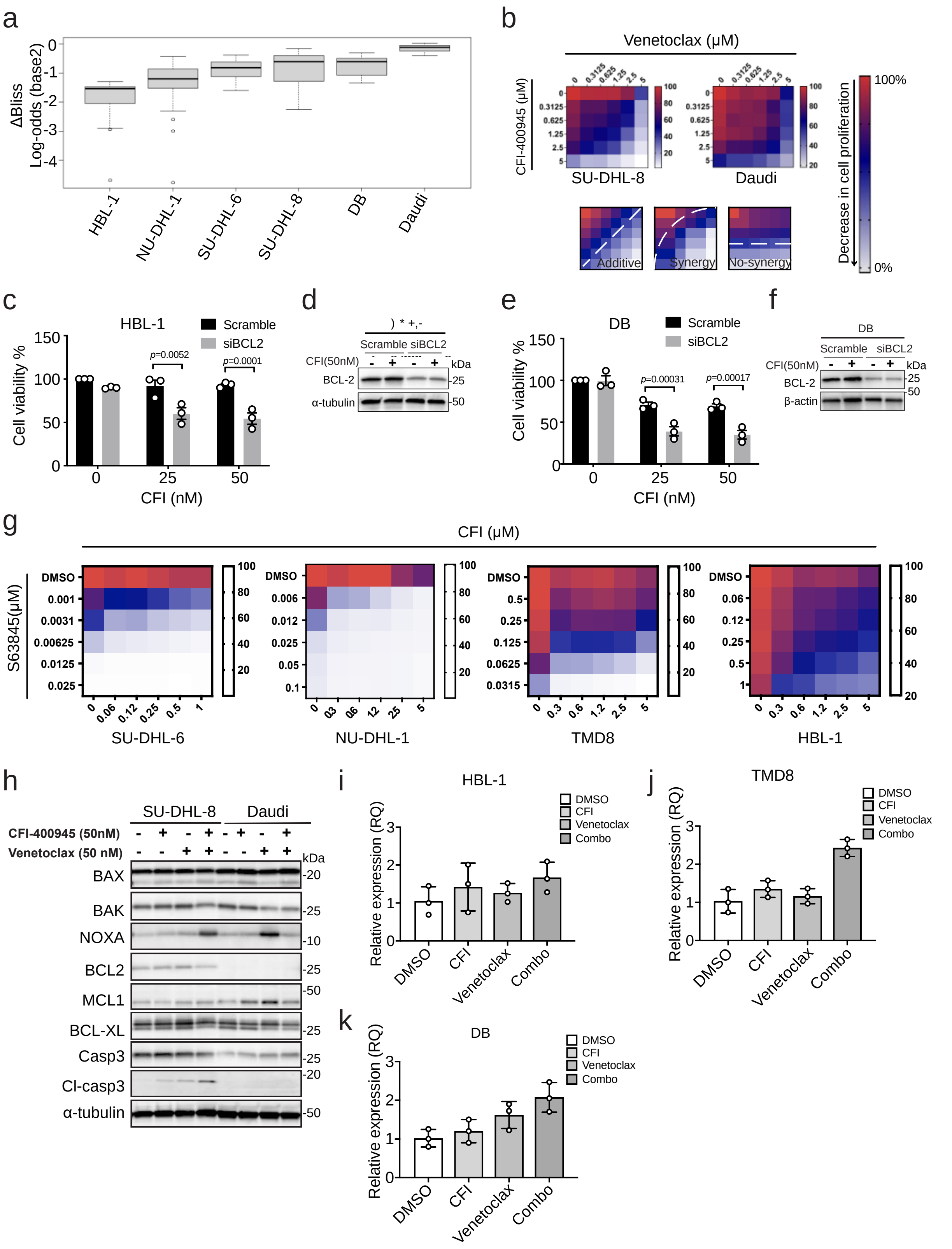

a
ΔBliss
Log-odds (base2)
0
-1
-2
-3
-4
HBL-1
NU-DHL-1
SU-DHL-6
SU-DHL-8
DB
Daudi
b
Venetoclax (μM)
CFI-400945 (μM)
SU-DHL-8
Daudi
Additive
Synergy
No-synergy
Decrease in cell proliferation
100%
0%
c
HBL-1
Scramble
siBCL2
Cell viability %
p=0.0052
p=0.0001
CFI (nM)
d
Scramble siBCL2
CFI(50nM)
kDa
BCL-2
α-tubulin
e
DB
Scramble
siBCL2
Cell viability %
p=0.00031
p=0.00017
CFI (nM)
f
DB
Scramble siBCL2
CFI(50nM)
kDa
BCL-2
β-actin
g
CFI (μM)
S63845(μM)
SU-DHL-6
NU-DHL-1
TMD8
HBL-1
h
SU-DHL-8
Daudi
CFI-400945 (50nM)
Venetoclax (50 nM)
kDa
BAX
BAK
NOXA
BCL2
MCL1
BCL-XL
Casp3
Cl-casp3
α-tubulin
i
HBL-1
Relative expression (RQ)
DMSO
CFI
Venetoclax
Combo
j
TMD8
Relative expression (RQ)
DMSO
CFI
Venetoclax
Combo
k
DB
Relative expression (RQ)
DMSO
CFI
Venetoclax
Combo

Supplementary Figure 4: Interaction between venetoclax and CFI in lymphoma cells. a) Delta bliss plot scores showing the effect of the combination of CFI and venetoclax in a panel of lymphoma cell lines. Maximum Log-odds = 0.1 (additive); minimum log-odds = −4.6 (synergy). Data represent mean ± SEM of three independent experiments for each cell line (n = 3). **b)** Drug matrix heatmap showing non-synergistic interaction between CFI and venetoclax in SU-DHL-8 and Daudi cell lines following drug treatment for 72h. As shown in the scheme (bellow), heatmap grids correspond to the mean of 3 independent experiments by the CellTiter-Glo assay. Cell viability is represented by different color gradients from red (0% inhibition) or blue to white (50-100% inhibition), scheme on the right. **c)** Cell viability assessed by CellTiter-Glo in HBL-1 cell line after BCL-2 siRNA (siBCL2) or scrambled siRNA control (Scramble). Cells were treated for 48 h with 25 nM or 50 nM CFI or with control (DMSO, 0.1%). Data represent mean ± SEM of three independent experiments (n = 3/condition). p values were calculated by two-way ANOVA followed by Tukey's multiple comparisons test. **d)** Western blots depicting the efficiency of BCL-2 knockdown in HBL-1 cells compared to the scrambled siRNA controls treated with CFI (50 nM) or Control DMSO (0.1%)**.** Representative blots from one of three experiments performed. **e)** Cell viability assessed by CellTiter-Glo in HBL-1 cell line after BCL-2 siRNA or scrambled siRNA control. Cells were treated for 48 h with 25 nM or 50 nM CFI or with control (DMSO, 0.1%). Data represent mean ± SEM of three independent experiments (n = 3/condition). p values were calculated by two-way ANOVA. **f)** Western blots depicting the efficiency of BCL-2 knockdown in DB cells compared to the scrambled siRNA controls. **g)** Drug matrix heatmap showing non-synergistic interaction between CFI and MCL-1 inhibitor, S63845 in lymphoma cell lines previously tested for CFI-venetoclax synergy (Figure 3a). Cell viability values are represented in a heatmap colored from red (0% inhibition) or blue to white (50-100% inhibition). Values represent the result of two independent experiments. **h)** Western blot analysis of anti- and pro-apoptotic Bcl-2 family proteins in SU-DHL-8 and Daudi cell lines after 72h of combinational treatment with indicated doses of CFI and venetoclax. Representative western blots from one of three

experiments performed. α-tubulin is shown to indicate equivalence of loading. **i)** Quantitative real time PCR analysis of BAX expression showing effect of 72h treatment with CFI (50nM), venetoclax (50nM) and their combination in HBL-1 **(j)** TMD8 and **(k)** DB cells compared with non-treated cells (DMSO 0,1%). Fold-change for RT-qPCR was normalized using GAPDH using the ΔΔCT method. Results are shown as mean ± SD of 3 technical replicates.

Immunoblots (panel **d, f and h**) result is the representative of at least three repeats. Statistics: *p* values for panels **c**, **e**, **i**, **j** and **k** were calculated by two-way ANOVA followed by Tukey's multiple comparisons test. Source data all panels are provided as a Source Data file.

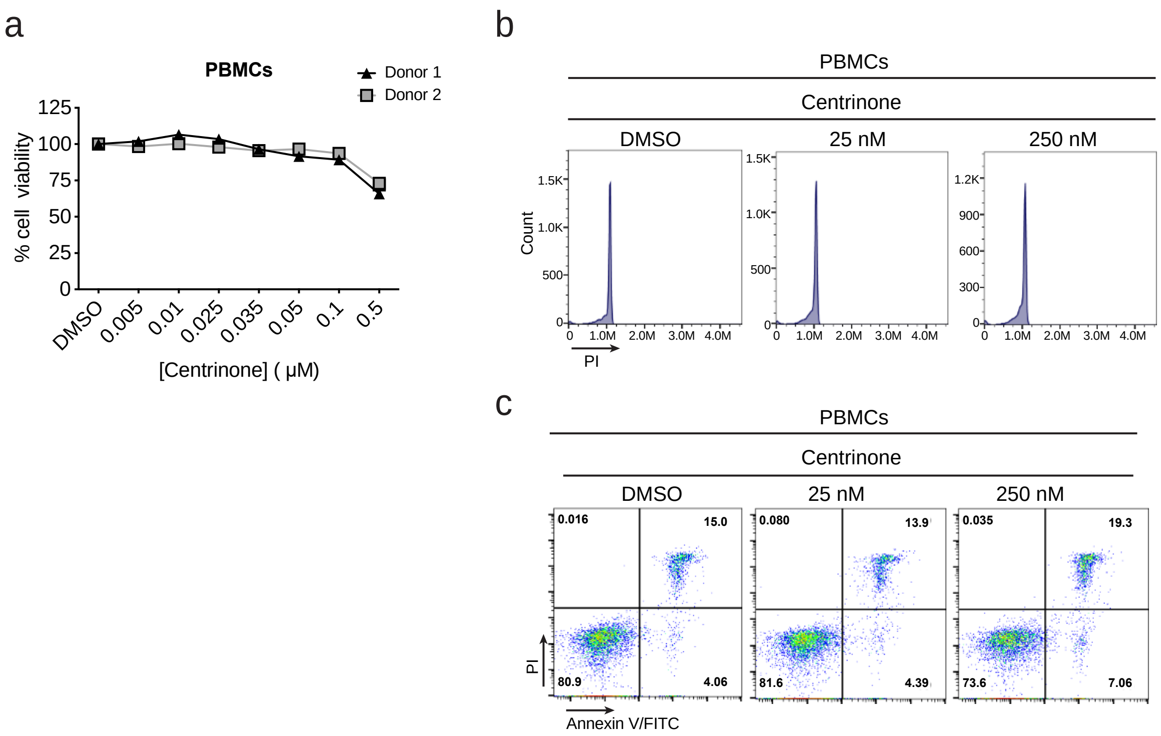

Supplementary Figure 5: Effect of centrinone combination on healthy PBMCs a) Cell viability of PBMCs isolated from whole blood from healthy donors measured by CellTiter-Glo after treatment with increasing concentrations of centrinone compared to DMSO (0.1%) for 72h. Values represents the mean±SEM of 2 replicates of independent donors, each time in triplicate. **b)**

Representative DNA histogram content- PI staining obtained by flow cytometry analysis in PBMCs treated with centrinone (25 and 250nM) or vehicle. **c)** Representative flow cytometry analysis of PBMC apoptosis staining with Annexin V-FITC/propidium iodide (PI) after treatment with centrinone (25 and 250nM) or DMSO (0,1%). Source data for panels **a** is provided as a Source Data file.

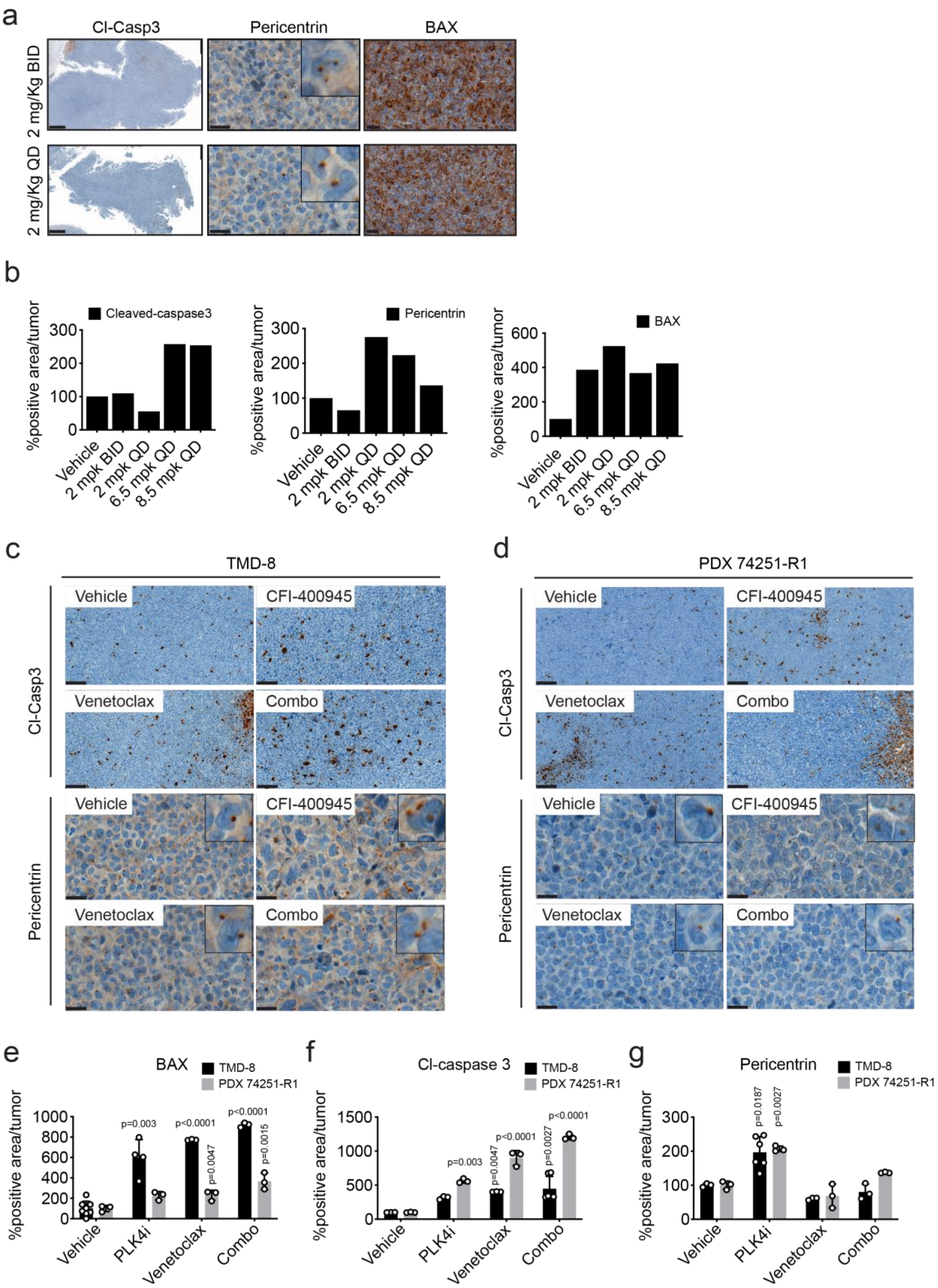
a
Cl-Casp3
Pericentrin
BAX
2 mg/Kg BID
2 mg/Kg QD
b
Cleaved-caspase3
Pericentrin
BAX
%positive area/tumor
Vehicle
2 mpk BID
2 mpk QD
6.5 mpk QD
8.5 mpk QD
c
TMD-8
d
PDX 74251-R1
Vehicle
CFI-400945
Venetoclax
Combo
Cl-Casp3
Pericentrin
e
BAX
f
Cl-caspase 3
g
Pericentrin
TMD-8
PDX 74251-R1
p=0.003
p<0.0001
p=0.0047
p=0.0015
p=0.0187
p=0.0027
PLK4i

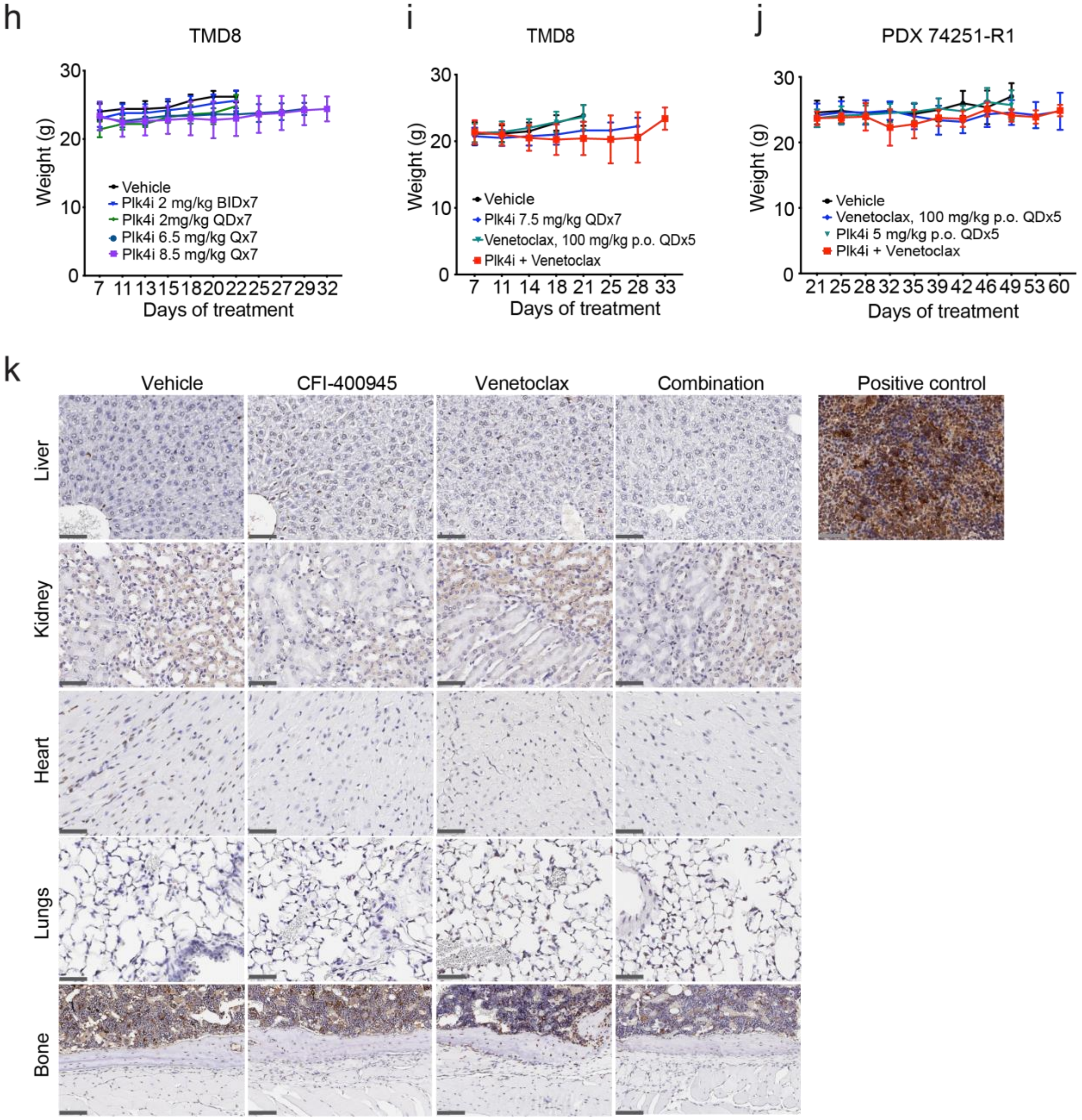

Supplementary Figure 6: Immunohistochemistry analysis and quantification of DLBCL mouse xenograft models. a) Cleaved (Cl) caspase 3, pericentrin and BAX levels obtained by immunohistochemical analysis of TMD-8 xenograft tumors following treatment with 2 mg/kg of CFI BID or QD or with vehicle. Representative non-necrotic areas from each tumor in each group are shown at a 100x magnification. Scale bars: Cl-Caspase, 500µm; Pericentrin, 20µm; BAX, 30 µm **b)** Bar graphs representing the percentage of total sectional area positive for cleaved caspase

3, Pericentrin and BAX staining in TMD8 xenografts of different treatment groups, represented in panel **a**. **c)** Immunohistochemical analysis of cleaved caspase-3 and pericentrin in TMD-8 xenograft and **d)** PDX-74251-R1 tumors following treatment with CFI, venetoclax, or in combination. Induction of cleaved caspase-3 was seen with venetoclax monotherapy, and this was enhanced by the addition of venetoclax. Pericentrin levels increased with CFI treatment and decrease to levels closed to normal with the addition of venetoclax. Representative non-necrotic areas from each tumor in each group are shown at a 100x magnification. Scale bars: Cl-Caspase, 100µm; BAX, 20µm **e)** Bar graphs representing the percentage of total sectional area positive for BAX, **f)** cleaved-caspase 3 and **g)** pericentrin staining in TMD-8 and PDX-74251-R1. Results are shown as mean ± SD (n = 3 biological samples/group). p values were calculated by two-way ANOVA followed by Tukey's multiple comparisons test. **h-j)** Animal weights during the indicated treatment studies remained constant. Data represents the mean percentage ± SD of initial values. [(**g**), *n*=5/group; (**h**) *n*=8,/group; (**i**), *n*=6/group)]. **k)** Apoptosis assessed by TUNEL assay of the liver, kidney, heart, lungs and bone of mice exposed to CFI, venetoclax, or the combination of both. There was no increase in TUNEL positive cells (brown) in any treatment group compared with the vehicle group. Tissues of the control and treatment groups were harvested three weeks following treatment. Three independent biological samples were assessed for each condition. Magnification, ×200. Scale bar, 100µm. Nuclei are stained in blue.

QD, once a day; BID, twice a day, p.o., oral administration. Statistics: *p* values for panels **e**, **f** and **g** were calculated by two-way ANOVA followed by Tukey's multiple comparisons test. Source data for panels **b**, **e**, **f**, **g**, **h**, **I** and **j** are provided as a Source Data file.

Supplementary Tables:

Supplementary table 1: BAX expression levels and TP53 mutation status in lymphoma cell lines (cBioPortal library). Grey cells mean absent data data; wt: Wild type.

		Treatment		
	TP53	CFI	Venetoclax	Combo
OCI-Ly19	wt	+++		
Z-138	wt	+++		
TMD8	wt	+		+++ (mRNA↑)
HBL-1	V157A	++	+	+++
Daudi	G266E	+		+
DB	R248W	+	++	+++ & (mRNA↑)
NU-DHL-1	R248Q	+	++	+++
SU-DHL-6	Y234C	++	+	++
SU-DHL-8	Y234N	+		+
SU-DHL-4	R273C	+		
PDX	R158H	+	++	++

Supplementary table 2: Mutational profile in DLBCL PDX model 74251-R1.
Oncogenic or likely oncogenic mutations are presented in bold (cBioPortal library).

Gene	Protein Change	Ref	Var	Mutation Type	Variant Type	Allele Freq (%)
**KMT2D**	**Q3601***	**G**	**A**	**Nonsense Mutation**	**SNP**	**65.7738095**
**CREBBP**	**Q935Hfs*63**	**C**	**-**	**Frame Shift Deletion**	**DEL**	**33.2727273**
**TP53**	**R158H**	**C**	**T**	**Missense Mutation**	**SNP**	**99.5145631**
**PIM1**	**G28A**	**G**	**C**	**Missense Mutation**	**SNP**	**62.568306**
**RB1**	**X287_splice**	**T**	**A**	**Splice Site**	**SNP**	**100**
ASMTL	D457N	C	T	Missense Mutation	SNP	49.3670886
BCL7A	R7*	C	T	Nonsense Mutation	SNP	67.1077505
CPS1	P271L	C	T	Missense Mutation	SNP	30.9392265
TRRAP	R3850H	G	A	Missense Mutation	SNP	34.2105263
MSH3	A61_P63dup	-	CCGCAGCGC	In Frame Insertion	INS	51.9455253
MYCL	G87E	C	T	Missense Mutation	SNP	37.3681567
EP300	Q2306R	A	G	Missense Mutation	SNP	67.8800857
FGFR4	R598Q	G	A	Missense Mutation	SNP	50.6646972
BTG1	P3H	G	T	Missense Mutation	SNP	31.1154599
TERT	D444G	T	C	Missense Mutation	SNP	99.3589744
BCL2	D31E	A	T	Missense Mutation	SNP	64.7058824
DTX1	V25G	T	G	Missense Mutation	SNP	29.8403194
MYCL	V139M	C	T	Missense Mutation	SNP	35.8024691
MYCL	A138S	C	A	Missense Mutation	SNP	35.4191263
MYCL	R106W	G	A	Missense Mutation	SNP	37.7751572
MYCL	Y50C	T	C	Missense Mutation	SNP	34.8262871
IRS2	S915N	C	T	Missense Mutation	SNP	38.2183908
CIITA	P1110H	C	A	Missense Mutation	SNP	65.3631285
EP300	I1185K	T	A	Missense Mutation	SNP	26.2711864
HIST1H1C	T96R	G	C	Missense Mutation	SNP	34.8726115
**MYCL**				**Amplification**		
**TNFRSF14**				**Deep Deletion**		
REL				Amplification		
XPO1				Amplification		
FANCL				Amplification		

Supplementary table 3: Rodent hematology, Acute treatment.

Group	Animal	NEU (x10^3/µL)	NEU %	LYM (x10^3/µL)	LYM %	MONO (x10^3/µL)	MONO %	EOS (x10^3/µL)	EOS %	BAS (x10^3/µL)	BAS %
**Vehicle**	An.1	0.98	9.10	16.10	46.10	50.60	17.70	34.90	13.10	1262.00	4.90
	An.2	0.58	7.43	12.80	37.50	50.50	17.20	34.00	13.10	1275.00	5.30
	An.3	1.57	8.23	14.30	41.60	50.50	17.40	34.50	13.60	1195.00	5.20
	**Mean**	**1.04**	**8.25**	**14.40**	**41.73**	**50.53**	**17.43**	**34.47**	**13.27**	**1244.00**	**5.13**
	**SD**	**0.50**	**0.84**	**1.65**	**4.30**	**0.06**	**0.25**	**0.45**	**0.29**	**42.93**	**0.21**
**CFI-400945**	An.1	0.75	8.28	14.40	42.10	50.90	17.40	34.10	13.50	1177.00	4.80
	An.2	0.86	8.23	14.00	41.40	50.20	17.00	33.80	13.20	1507.00	5.10
	An.3	1.10	8.46	14.60	43.00	50.90	17.30	34.00	13.70	1298.00	5.20
	**Mean**	**0.90**	**8.32**	**14.33**	**42.17**	**50.67**	**17.23**	**33.97**	**13.47**	**1327.33**	**5.03**
	**SD**	**0.18**	**0.12**	**0.31**	**0.80**	**0.40**	**0.21**	**0.15**	**0.25**	**166.94**	**0.21**
**Venetoclax**	An.1	1.13	8.58	14.70	44.00	51.20	17.10	33.40	13.10	1116.00	5.30
	An.2	1.36	8.67	15.10	43.90	50.60	17.40	34.30	13.60	1052.00	5.30
	An.3	0.85	8.94	15.70	45.70	51.10	17.60	34.40	13.50	1075.00	4.90
	**Mean**	**1.11**	**8.73**	**15.17**	**44.53**	**50.97**	**17.37**	**34.03**	**13.40**	**1081.00**	**5.17**
	**SD**	**0.26**	**0.19**	**0.50**	**1.01**	**0.32**	**0.25**	**0.55**	**0.26**	**32.42**	**0.23**
**Combo**	An.1	0.80	8.22	14.30	41.50	50.50	17.40	34.50	13.50	1472.00	5.10
	An.2	0.50	8.23	14.60	41.20	50.00	17.70	35.40	13.40	1286.00	5.10
	An.3	0.99	6.58	11.80	33.40	50.80	17.90	35.30	13.80	1192.00	5.30
	**Mean**	**0.76**	**7.68**	**13.57**	**38.70**	**50.43**	**17.67**	**35.07**	**13.57**	**1316.67**	**5.17**
	**SD**	**0.25**	**0.95**	**1.54**	**4.59**	**0.40**	**0.25**	**0.49**	**0.21**	**142.50**	**0.12**

Group	Animal	NEU (x10^3/µL)	NEU %	LYM (x10^3/µL)	LYM %	MONO (x10^3/µL)	MONO %	EOS (x10^3/µL)	EOS %	BAS (x10^3/µL)	BAS %
**Vehicle**	An.1	0.82	83.70	0.02	2.10	0.11	0.03	11.00	0.00	0.70	2.50
	An.2	0.44	74.20	0.05	10.00	0.08	0.01	12.60	0.00	0.60	2.60
	An.3	1.29	82.10	0.04	2.80	0.20	0.04	12.50	0.00	0.10	2.50
	**Mean**	**0.85**	**80.00**	**0.04**	**4.97**	**0.13**	**0.03**	**12.03**	**0.00**	**0.47**	**2.53**
	**SD**	**0.43**	**5.09**	**0.02**	**4.37**	**0.06**	**0.02**	**0.90**	**0.00**	**0.32**	**0.06**
**CFI-400945**	An.1	0.57	75.70	0.04	4.20	0.12	0.02	15.80	0.00	0.80	3.50
	An.2	0.70	81.10	0.01	1.60	0.13	0.02	14.20	0.00	0.50	2.60
	An.3	0.85	76.60	0.01	1.40	0.21	0.03	19.10	0.00	0.10	2.80
	**Mean**	**0.71**	**77.80**	**0.02**	**2.40**	**0.15**	**0.02**	**16.37**	**0.00**	**0.47**	**2.97**
	**SD**	**0.14**	**2.89**	**0.02**	**1.56**	**0.05**	**0.01**	**2.50**	**0.00**	**0.35**	**0.47**
**Venetoclax**	An.1	0.88	77.80	0.01	1.20	0.18	0.06	15.80	0.00	0.30	4.90
	An.2	1.14	83.70	0.05	3.50	0.15	0.02	10.40	0.00	0.60	1.80
	An.3	0.70	82.40	0.03	3.30	0.10	0.02	11.80	0.00	0.70	1.80
	**Mean**	**0.91**	**81.30**	**0.03**	**2.67**	**0.14**	**0.03**	**12.67**	**0.00**	**0.53**	**2.83**
	**SD**	**0.22**	**3.10**	**0.02**	**1.27**	**0.04**	**0.02**	**2.80**	**0.00**	**0.21**	**1.79**
**Combo**	An.1	0.63	78.00	0.05	7.00	0.10	0.02	12.10	0.00	0.10	2.80
	An.2	0.43	84.50	0.04	7.20	0.03	0.00	5.30	0.00	1.20	1.80
	An.3	0.75	75.50	0.04	4.00	0.18	0.02	17.60	0.00	0.00	2.90
	**Mean**	**0.60**	**79.33**	**0.04**	**6.07**	**0.10**	**11.67**	**0.01**	**2.50**	**0.00**	**0.43**
	**SD**	**0.16**	**4.65**	**0.01**	**1.79**	**0.08**	**6.16**	**0.01**	**0.61**	**0.00**	**0.67**

Supplementary table 4: Rodent hematology, final treatment.

Group	Animal no.	WBC (x10³/µL)	RBC (x10⁶/µL)	HGB (g/dL)	HCT (x10³/µL)	MCV %	MCH (pg)	MCHC (g/dL)	RDW %	PLT (x10³/µL)	MPV (fL)
**Vehicle**	An.1	1.31	8.46	13.90	42.20	49.80	16.40	33.00	13.00	1250.00	4.80
	An.2	0.83	7.96	13.00	39.10	49.10	16.30	33.20	13.10	1339.00	4.90
	An.3	0.91	7.29	11.90	35.80	49.10	16.30	33.20	13.90	1507.00	5.00
	**Mean**	**1.02**	**7.90**	**12.93**	**39.03**	**49.33**	**16.33**	**33.13**	**13.33**	**1365.33**	**4.90**
	**SD**	**0.26**	**0.59**	**1.00**	**3.20**	**0.40**	**0.06**	**0.12**	**0.49**	**130.51**	**0.10**
**CFI-400945**	An.1	0.97	8.13	13.40	40.30	49.60	16.50	33.20	13.10	1283.00	4.90
	An.2	0.94	8.06	13.50	40.00	49.60	16.70	33.70	12.90	1148.00	5.00
	An.3	0.94	8.01	13.60	39.90	49.90	16.90	34.00	13.50	1198.00	5.00
	**Mean**	**0.95**	**8.07**	**13.50**	**40.07**	**49.70**	**16.70**	**33.63**	**13.17**	**1209.67**	**4.97**
	**SD**	**0.02**	**0.06**	**0.10**	**0.21**	**0.17**	**0.20**	**0.40**	**0.31**	**68.25**	**0.06**
**Venetoclax**	An.1	1.56	8.25	13.50	41.10	49.90	16.40	32.90	13.50	1372.00	5.10
	An.2	1.01	8.34	14.30	42.30	50.70	17.10	33.70	13.80	1269.00	5.00
	An.3	1.52	8.68	14.30	43.20	49.80	16.50	33.10	13.60	1407.00	5.10
	**Mean**	**1.36**	**8.42**	**14.03**	**42.20**	**50.13**	**16.67**	**33.23**	**13.63**	**1349.33**	**5.07**
	**SD**	**0.31**	**0.23**	**0.46**	**1.05**	**0.49**	**0.38**	**0.42**	**0.15**	**71.74**	**0.06**
**Combo**	An.1	1.31	8.74	14.60	44.50	50.90	16.70	32.80	13.40	1211.00	4.80
	An.2	0.97	8.27	13.50	41.80	50.50	16.30	32.40	13.10	1344.00	4.90
	An.3	1.09	8.36	13.40	41.60	49.80	16.00	32.10	13.60	1536.00	4.90
	**Mean**	**1.12**	**8.46**	**13.83**	**42.63**	**50.40**	**16.33**	**32.43**	**13.37**	**1363.67**	**4.87**
	**SD**	**0.17**	**0.25**	**0.67**	**1.62**	**0.56**	**0.35**	**0.35**	**0.25**	**163.39**	**0.06**
Reference range		0.46 - 2.70	7.22 - 9.15	12.06 - 15.57	37.04 - 47.72	49.98 - 53.64	16.28 - 17.48	31.46 - 33.71	12.99 - 15.61	1044 - 1531	4.69 - 5.32

Group	Animal	NEU (x10³/µL)	NEU %	LYM (x10³/µL)	LYM %	MONO (x10³/µL)	MONO %	EOS (x10³/µL)	EOS %	BAS (x10³/µL)	BAS %
**Vehicle**	An.1	1.12	84.90	0.02	1.40	0.17	12.80	0.00	0.50	0.00	0.40
	An.2	0.71	84.90	0.01	1.50	0.10	11.30	0.01	1.70	0.00	0.60
	An.3	0.75	81.60	0.00	0.90	0.15	15.80	0.01	1.70	0.00	0.00
	**Mean**	**0.86**	**83.80**	**0.01**	**1.27**	**0.14**	**13.30**	**0.01**	**1.30**	**0.00**	**0.33**
	**SD**	**0.23**	**1.91**	**0.01**	**0.32**	**0.04**	**2.29**	**0.01**	**0.69**	**0.00**	**0.31**
**CFI-400945**	An.1	0.76	78.30	0.00	0.40	0.19	19.40	0.02	1.90	0.00	0.00
	An.2	0.73	77.60	0.00	0.40	0.19	19.40	0.02	2.20	0.00	0.40
	An.3	0.67	71.10	0.01	1.40	0.24	25.10	0.02	1.50	0.00	0.90
	**Mean**	**0.72**	**75.67**	**0.00**	**0.73**	**0.21**	**21.30**	**0.02**	**1.87**	**0.00**	**0.43**
	**SD**	**0.05**	**3.97**	**0.01**	**0.58**	**0.03**	**3.29**	**0.00**	**0.35**	**0.00**	**0.45**
**Venetoclax**	An.1	1.10	69.90	0.03	2.20	0.33	20.90	0.09	6.30	0.01	0.70
	An.2	0.74	73.10	0.02	2.70	0.21	20.00	0.04	3.70	0.00	0.50
	An.3	1.19	78.20	0.00	0.10	0.30	19.50	0.03	1.80	0.00	0.40
	**Mean**	**1.01**	**73.73**	**0.02**	**1.67**	**0.28**	**20.13**	**0.05**	**3.93**	**0.00**	**0.53**
	**SD**	**0.24**	**4.19**	**0.02**	**1.38**	**0.06**	**0.71**	**0.03**	**2.26**	**0.01**	**0.15**
**Combo**	An.1	0.92	69.70	0.01	1.00	0.34	25.60	0.04	3.20	0.00	0.50
	An.2	0.76	77.40	0.00	0.60	0.19	19.60	0.02	1.90	0.00	0.50
	An.3	0.77	70.40	0.00	0.70	0.27	24.20	0.05	4.70	0.00	0.00
	**Mean**	**0.82**	**72.50**	**0.00**	**0.77**	**0.27**	**23.13**	**0.04**	**3.27**	**0.00**	**0.33**
	**SD**	**0.09**	**4.26**	**0.01**	**0.21**	**0.08**	**3.14**	**0.02**	**1.40**	**0.00**	**0.29**
Reference range		0.34 - 2.19	71.09 - 88.40	0.00 - 0.04	0.00 - 2.62	0.04 - 0.44	7.46 - 22.63	0.00 - 0.12	0.39 - 6.83	0.00 - 0.03	0.0 - 1.5
