# Supplementary material for: Synthetic lethality of drug-induced polyploidy and BCL-2 inhibition in lymphoma
Source: Nat Commun. 2023 Mar 18;14:1522. doi: 10.1038/s41467-023-37216-2 (PMC10024740; doi:10.1038/s41467-023-37216-2)
Supplement: Supplementary file 2 — Reporting Summary [file 41467_2023_37216_MOESM2_ESM.pdf]

nature portfolio

Corresponding author(s): Hans-Guido Wendel; Anas Younes

Last updated by author(s): Feb 8, 2023

Reporting Summary

Nature Portfolio wishes to improve the reproducibility of the work that we publish. This form provides structure for consistency and transparency in reporting. For further information on Nature Portfolio policies, see our Editorial Policies and the Editorial Policy Checklist.

Statistics

For all statistical analyses, confirm that the following items are present in the figure legend, table legend, main text, or Methods section.

n/a	Confirmed	
☐	☒	The exact sample size (n) for each experimental group/condition, given as a discrete number and unit of measurement
☐	☒	A statement on whether measurements were taken from distinct samples or whether the same sample was measured repeatedly
☐	☒	The statistical test(s) used AND whether they are one- or two-sided<br>*Only common tests should be described solely by name; describe more complex techniques in the Methods section.*
☒	☐	A description of all covariates tested
☒	☐	A description of any assumptions or corrections, such as tests of normality and adjustment for multiple comparisons
☐	☒	A full description of the statistical parameters including central tendency (e.g. means) or other basic estimates (e.g. regression coefficient) AND variation (e.g. standard deviation) or associated estimates of uncertainty (e.g. confidence intervals)
☐	☒	For null hypothesis testing, the test statistic (e.g. F, t, r) with confidence intervals, effect sizes, degrees of freedom and P value noted<br>*Give P values as exact values whenever suitable.*
☒	☐	For Bayesian analysis, information on the choice of priors and Markov chain Monte Carlo settings
☒	☐	For hierarchical and complex designs, identification of the appropriate level for tests and full reporting of outcomes
☒	☐	Estimates of effect sizes (e.g. Cohen's d, Pearson's r), indicating how they were calculated

Our web collection on statistics for biologists contains articles on many of the points above.

Software and code

Policy information about availability of computer code

Data collection	Western blot images were acquired by using the Bio-Rad Image Lab 6.1 Software for Mac. Lumisnescence data was collected using Kaleido version 1.2. Flow cytometry data was collected using CellQuest Pro Version 6. 0 . Mutation information was collected using cBioPortal (MSKCC)
Data analysis	Immunofluorescence images were processed with Airyscan and converted into .ims files to create 3d images using Imaris 10.0 (BIT PLANE, Switzerland). Quantification of these images was performed using ImageJ V1.53 and the 3dMaximaFinder plugin was used to detect and count the individual centrosomes within each nucleus based on their relative maximum intensity. Data analysis, statistical testing and visualization were conducted in Graph pad Prism (v.7). Flow cytometry data was analyzed with FlowJo (Tree Star) v10. QuantStudio Software vl.7.2 was used to analyze Q-PC R data.

For manuscripts utilizing custom algorithms or software that are central to the research but not yet described in published literature, software must be made available to editors and reviewers. We strongly encourage code deposition in a community repository (e.g. GitHub). See the Nature Portfolio guidelines for submitting code & software for further information.

Data

Policy information about availability of data

All manuscripts must include a data availability statement. This statement should provide the following information, where applicable:

- Accession codes, unique identifiers, or web links for publicly available datasets
- A description of any restrictions on data availability
- For clinical datasets or third party data, please ensure that the statement adheres to our policy

The datasets generated during and/or analyzed during the current study are available are provided within the Article, Supplementary information or Source Data file.

Human research participants

Policy information about studies involving human research participants and Sex and Gender in Research.

Reporting on sex and gender	N/A
Population characteristics	N/A
Recruitment	N/A
Ethics oversight	N/A

Note that full information on the approval of the study protocol must also be provided in the manuscript.

Field-specific reporting

Please select the one below that is the best fit for your research. If you are not sure, read the appropriate sections before making your selection.

[x] Life sciences [ ] Behavioural & social sciences [ ] Ecological, evolutionary & environmental sciences

For a reference copy of the document with all sections, see nature.com/documents/nr-reporting-summary-flat.pdf

Life sciences study design

All studies must disclose on these points even when the disclosure is negative.

Sample size	The sample size was chosen on the basis of prior studies that showed significant effects with similar sample sizes. For in vitro cell viability assays and Western blots and Flow cytometry assays we aimed for a minimum of 3 independent experiments peer group to allow for basic statistical inference. Or 2 independent experiments when statistical inference was not required. For in vivo studies, at least five mice per group were included in order to achieve an alpha value of 0.8.
Data exclusions	Data were not excluded from analysis.
Replication	All attempts of replication were successful and gave similar results. The number of independent biological repeats performed for each experiment are indicated through the manuscript main content, methods, and figure legends. Experiments were repeated at least twice by different scientists in the lab. Animal experiments were performed on at least n=5 mice per group for each experiment.
Randomization	No randomization. This is not relevant because the samples are derived from defined groups. All samples were treated in the same way to decrease the variability.
Blinding	Data collection/generation and analysis was not blinded to the operator for the different discovery experiments. Quantifications were performed using computational pipeline applied equally to all conditions and replicates for a given group.

Reporting for specific materials, systems and methods

We require information from authors about some types of materials, experimental systems and methods used in many studies. Here, indicate whether each material, system or method listed is relevant to your study. If you are not sure if a list item applies to your research, read the appropriate section before selecting a response.

Materials & experimental systems

n/a	Involved in the study
☐	☒ Antibodies
☐	☒ Eukaryotic cell lines
☒	☐ Palaeontology and archaeology
☐	☒ Animals and other organisms
☒	☐ Clinical data
☒	☐ Dual use research of concern

Methods

n/a	Involved in the study
☒	☐ ChIP-seq
☐	☒ Flow cytometry
☒	☐ MRI-based neuroimaging

Antibodies

Antibodies used	Antibodies supplied by Abcam: NOXA (cat. ab13654, dilution 1:1000), STIL (ab89314, dilution 1:1000) and pericentrin (ab4448, dilution, 1:250). Antibodies supplied by Cell signaling Technology: BAX (#2774S, dilution, 1:1000), BAK (#12105S, dilution, 1:1000), Cleaved Caspase-3 (#9661, dilution, 1:1000), Caspase-3 (#9662, dilution, 1:1000), BCL-2 (#4223S, dilution, 1:1000), BCL-xL (#2764S, dilution, 1:1000), MCL-1 (#4572S, dilution, 1:1000), PUMA (#12450S, dilution, 1:1000), alpha-tubulin (# 3873, dilution, 1:10000). Antibodies supplied by Sigma: Beta-Actin (#5316, dilution, 1:10000). CEP110 was purchased from EMD Millipore (MABT1354, dilution 1:250). PLK4 (#12952-1-AP, dilution 1:1000) was purchased from Proteintech Antibodies supplied by Vector Labs: Biotinylated rabbit anti-goat IgG (Cat# BA-5000, dilution, 1:10000) and Biotinylated goat anti-rabbit IgG (Cat# PK-6101, dilution, 1:10000)
Validation	All antibodies are commercially available and have been validated for the indicated application by the manufacturers

Eukaryotic cell lines

Policy information about cell lines and Sex and Gender in Research

Cell line source(s)	REC-1, JeKo-1, Z-138, Mino, JVM-2, JVM-13, Raji, EBl, Daudi, Ramos and CA46 were obtained from ATCC (American Type Culture Collection). SU-DHL-4, SU-DHL-6, SU-DHL-8, SU-DHL-10 OCY-Ly19, DB, NU-DHL-1, U-2973, OCI-Ly-3, U-2932, Rl-1, OCI-Ly-10), L-428, HDLM-2 and KM-H2, MAVER-1 were obtained from the DSMZ-German Collection of Microorganisms and Cell Cultures. The cell lines BJAB, HBL-1 and TMD8, SUP-M2, SU-DHL-1 and KARPAS-299 are not commercial available and were provided by Dr. R.E. Davis (MD Anderson Cancer Center, Houston, TX. Z138-cas9 and OCI-Ly19-cas 9 cells were made and validated ate MSKCC exclusively for this study.
Authentication	Cell lines were autheniticaed by STR analysis at the Integrated Genomic Operation Core Facility at Memorial Sloan KEttering Cancer Center, New York, NY.
Mycoplasma contamination	Cells were routinely tested and confirmed negative for mycoplasma in house using the mycoplasma detection kit available from Lonza Biosciences (cat.#LT07-218) according to manufacturer's instructions
Commonly misidentified lines (See ICLAC register)	No commonly misidentified cell lines were used in this study.

Animals and other research organisms

Policy information about studies involving animals; ARRIVE guidelines recommended for reporting animal research, and Sex and Gender in Research

Laboratory animals	6-week old female NSG mice (NOD SCID Gamma) were used for the xenograft studies. Housing conditions are provided in the methods section.
Wild animals	This study did not involve wild animals.
Reporting on sex	We performed experiments with cell lines and patient samples that represent both sexes
Field-collected samples	This study did not involve samples collected from the field.
Ethics oversight	All animal studies were reviewed and approved by the Institutional Animal Care and Use Committee (IACUC) at Jubilant BioSys, Memorial Sloan Kettering Cancer Center (MSKCC) and at START, Center for Cancer Care. All mice were maintained in accordance with the guidelines of Association for Assessment and Accreditation of Laboratory Animal Care International (AAALAC) on the care, welfare, and treatment of laboratory animals and all experiments were conducted under approved protocols from Institutional Animal Care and Use Committees (IACUC).

Note that full information on the approval of the study protocol must also be provided in the manuscript.

Flow Cytometry

Plots

Confirm that:

- [x] The axis labels state the marker and fluorochrome used (e.g. CD4-FITC).
- [x] The axis scales are clearly visible. Include numbers along axes only for bottom left plot of group (a 'group' is an analysis of identical markers).
- [x] All plots are contour plots with outliers or pseudocolor plots.
- [x] A numerical value for number of cells or percentage (with statistics) is provided.

Methodology

Sample preparation	Cells were collected from 24 or 6 well plates, washed twice with PBS, resuspended in flow cytometry sorting buffer (PBS supplemented with 1 mM EDTA, 25 mM HEPES and 0.5% FBS) and then stained for analysis. All details are mentioned in the Methods section.
Instrument	BD FACSCalibur
Software	CellQuest Pro Version 6.0
Cell population abundance	Greater than 10,000 events were acquired.
Gating strategy	Samples were initially gated using forward scatter and side scatter to identify events corresponding to cells, and then using forward scatter height vs. area to enrich for single cells. Doublets were excluded by gating out high FL3- W (width) cells. Alive cells were selected by negativity for viability dye.

- [x] Tick this box to confirm that a figure exemplifying the gating strategy is provided in the Supplementary Information.
