# Supplementary material for: Synthetic lethality of drug-induced polyploidy and BCL-2 inhibition in lymphoma
Source: Nat Commun. 2023 Mar 18;14:1522. doi: 10.1038/s41467-023-37216-2 (PMC10024740; doi:10.1038/s41467-023-37216-2)
Supplement: Supplementary file 3 — Description of Additional Supplementary Files [file 41467_2023_37216_MOESM3_ESM.pdf]

Description of Additional Supplementary Files

File Name: Supplementary Data 1.
Description: Rodent chemistry panel, acute treatment values.
Description: Rodent chemistry panel, acute treatment values with respective reference ranges.

File Name: Supplementary Data 2.
Description: Rodent chemistry panel, final treatment values.
Description: Rodent chemistry panel, final treatment values with respective reference ranges.
